# Somatostatin receptors (SSTR1-5) on inhibitory interneurons in the barrel cortex

**DOI:** 10.1007/s00429-019-02011-7

**Published:** 2019-12-23

**Authors:** Agnieszka Lukomska, Grzegorz Dobrzanski, Monika Liguz-Lecznar, Malgorzata Kossut

**Affiliations:** Laboratory of Neuroplasticity, Nencki Institute of Experimental Biology of Polish Academy of Sciences, Warsaw, Poland

**Keywords:** Somatostatin receptors, PV interneurons, SST interneurons, VIP interneurons, Barrel cortex, Somatosensory cortex

## Abstract

Inhibitory interneurons in the cerebral cortex contain specific proteins or peptides characteristic for a certain interneuron subtype. In mice, three biochemical markers constitute non-overlapping interneuron populations, which account for 80–90% of all inhibitory cells. These interneurons express parvalbumin (PV), somatostatin (SST), or vasoactive intestinal peptide (VIP). SST is not only a marker of a specific interneuron subtype, but also an important neuropeptide that participates in numerous biochemical and signalling pathways in the brain via somatostatin receptors (SSTR1-5). In the nervous system, SST acts as a neuromodulator and neurotransmitter affecting, among others, memory, learning, and mood. In the sensory cortex, the co-localisation of GABA and SST is found in approximately 30% of interneurons. Considering the importance of interactions between inhibitory interneurons in cortical plasticity and the possible GABA and SST co-release, it seems important to investigate the localisation of different SSTRs on cortical interneurons. Here, we examined the distribution of SSTR1-5 on barrel cortex interneurons containing PV, SST, or VIP. Immunofluorescent staining using specific antibodies was performed on brain sections from transgenic mice that expressed red fluorescence in one specific interneuron subtype (PV-Ai14, SST-Ai14, and VIP-Ai14 mice). SSTRs expression on PV, SST, and VIP interneurons varied among the cortical layers and we found two patterns of SSTRs distribution in L4 of barrel cortex. We also demonstrated that, in contrast to other interneurons, PV cells did not express SSTR2, but expressed other SSTRs. SST interneurons, which were not found to make chemical synapses among themselves, expressed all five SSTR subtypes.

**Electronic supplementary material:**

The online version of this article (10.1007/s00429-019-02011-7) contains supplementary material, which is available to authorized users.

## Introduction

GABAergic interneurons, although not very abundant, constitute the most heterogeneous group of cortical neurons with respect to morphology, electrophysiological characteristics, and pattern of gene expression (DeFelipe 1993; Markram et al. 2004; Petilla Interneuron Nomenclature et al. 2008; Szentagothai 1978). All of these characteristics can be used for interneuron classification; however, one of the most popular criteria used is the biochemical profile of GABAergic neurons. Several substances that co-localise with GABA in interneurons have been identified. Approximately 40% of the inhibitory interneurons in the sensory cortex express parvalbumin (PV INs), 30% express somatostatin (SST INs), 30% express serotonin receptor 3a (5HT3aR), and 40% of 5HT3aR-neurons express vasoactive intestinal peptide (VIP INs) (Lee et al. 2010; Tremblay et al. 2016). In mice, neurons containing PV, SST, and VIP constitute non-overlapping categories that account for 80–90% of all inhibitory cells (Pfeffer et al. 2013; Rudy et al. 2011). These neurons also tend to exhibit specific expression patterns in the mouse neocortex: VIP INs are located primarily in L2/3, whereas SST INs and PV INs are found in layers 2–6 (Neske et al. 2015; Wall et al. 2016).

In cortical microcircuits, each subpopulation has a propensity for controlling particular domains of glutamatergic principal cells with specific timing. Fast spiking PV INs regulate the excitability and the generation of the action potential in pyramidal cells (PCs) and mainly target the perikarya, while SST INs control signal integration by targeting apical dendrites (Kawaguchi and Kubota 1997). VIP INs, on the other hand, do not target principal cells, but rather selectively regulate the activity of other interneuron subpopulations, mostly SST INs (Pfeffer 2014). The observation that SST INs form more synapses with other interneurons than with PCs (Cottam et al. 2013), suggests an important role of SST INs in the modulation of VIP INs and PV INs firing (Artinian and Lacaille 2018; Feldmeyer et al. 2018; Karnani et al. 2016a). However, SST neurons do not inhibit each other (Pfeffer et al. 2013; Karnani et al. 2016b). Controlling interneurons is of fundamental importance as the modulation of synaptic integration and network activity is crucial for the regulation of information processing.

Many studies have shown that the different subtypes of interneurons display different activation patterns in learned behaviours and characteristic behavioural states, and they are important players in neuroplasticity (Gentet et al. 2012; Hensch 2005; Katona et al. 2014; Lee et al. 2012; Nys et al. 2015; Pinto and Dan 2015; Sachidhanandam et al. 2016; Schneider et al. 2014). SST INs have been found to be involved in the cellular mechanisms of learning (Adler et al. 2019; Chen et al. 2015; Cichon and Gan 2015; Kato et al. 2015; Letzkus et al. 2011; Lovett-Barron et al. 2014; McKay et al. 2013; Pi et al. 2013; Stefanelli et al. 2016; Wolff et al. 2014) and cortical plasticity (Fu et al. 2015; Khan et al. 2018; Scheyltjens and Arckens 2016). Our studies of learning-dependent plasticity in the barrel cortex point to the involvement of SST containing GABAergic neurons in the mechanism underlying the development of plastic changes (Cybulska-Klosowicz et al. 2013).

Somatostatin is not merely a marker of a group of inhibitory interneurons; it is a neurotransmitter and neuromodulator as well (Patel 1999; Viollet et al. 2008). Its action is inhibitory and the dampening of SST receptors (SSTRs), especially SSTR2 and SSTR4, is considered a possible anti-epileptic treatment (Moneta et al. 2002; Tallent and Qiu 2008). Synaptically released SST may contribute to the augmentation of inhibitory action of GABA probably via a co-release mechanism (Katona et al. 2014; Leresche et al. 2000; Twery and Gallagher 1990; Gonchar et al. 2001), although this mechanism was not examined in sensory cortex. SST is stored in presynaptic terminals in dense-core vesicles therefore its release may be slower than classical neurotransmitter (Baraban and Tallent 2004). Moreover, as a peptide, SST is more likely to be released by high-frequency firing (van den Pol 2012); consequently, somatostatin may act slower and its effects may be longer-lasting than those of GABA. SST may also act more broadly, activating extrasynaptic receptors (Dournaud et al. 1998). Released SST acts on five types of receptors (SSTR1-5), which are G protein-coupled receptors (GPCRs). SSTR1 acts presynaptically, SSTR2, 4, and 5 act postsynaptically, and SSTR3 acts extrasynaptically (Günther et al., 2018). The presynaptic action of SST can decrease neurotransmitter release and diminish the input to PCs, while postsynaptically, SST most often hyperpolarises the target neuron, inducing a slow, but long-lasting inhibition (Momiyama and Zaborszky 2006).

We decided to examine and describe the layer-specific localisation of SSTR subtypes on PV, VIP, and SST inhibitory interneurons in the mouse cortex. We choose the region of the barrel cortex, which contains a representation of the whiskers and is somatotopically arranged, forming a pattern of so-called barrels in L4 that reflects the whisker arrangement on the snout (Woolsey and Van der Loos 1970). This cortical region is of primary interest in sensory processing, sensorimotor integration and neuroplasticity studies, and the interneurons of the barrel cortex, as well as their connectivity, have been studied in considerable detail (for a recent review, see Feldmeyer et al. 2013; Petersen 2007). Nevertheless, the SSTRs distribution in particular cortical layers and the localisation of SSTRs on particular types of interneurons has not been investigated in detail thus far.

We found two patterns of SSTR distribution in the mouse barrel cortex. Specifically, we observed that, in contrast to other interneurons, PV cells did not express SSTR2, while VIP INs and SST INs expressed all five SSTR subtypes.

## Material and methods

### Animals

Transgenic homozygous PV-Cre, SST-Cre, VIP-Cre mice were crossed with homozygous Ai14 mice line (stock no: 008069, 013044, 010908 and 007914, respectively) to obtain F1, heterozygous animals (PV-Ai14, SST-Ai14, or VIP-Ai14) expressing red fluorescent protein, tdTomato, following Cre-mediated recombination. Six-week-old male transgenic F1 animals and wild type C57BL/6 were used in the study. Transgenic mice were acquired from The Jackson Laboratory, USA and bred in the Animal Facility of the Nencki Institute of Experimental Biology PAS (Warsaw, Poland). Genotyping of transgenic mice was performed according to protocols provided by The Jackson Laboratory. The animals were housed under a 12-h light/dark cycle in a temperature and humidity-controlled room with ad libitum access to standard laboratory chow and water. All procedures were performed in accordance with the local ethical committee.

### Antibodies and blocking peptides

The primary antibodies used in this study were as follows: rabbit anti-SSTR1 polyclonal antibody (1:400, catalogue no. ASR-001, Alomone Labs), rabbit anti-SSTR2 monoclonal antibody (1:2000, catalogue no. ab134152, Abcam), rabbit anti-SSTR3 polyclonal antibody (1:300, catalogue no. ASR-003, Alomone Labs), rabbit anti-SSTR4 polyclonal antibody (1:1000, catalogue no. ab28578, Abcam), and rabbit anti-SSTR5 polyclonal antibody (1:1000, catalogue no. ASR-005, Alomone Labs).

A goat anti-rabbit polyclonal immunoglobulin G (IgG)-conjugated Alexa Fluor 488 antibody was used (catalogue no. 711-545-152, Jackson ImmunoResearch Europe Ltd) as a secondary antibody.

The blocking peptides used in this research to confirm antibody specificity were as follows: SSTR1 control peptide (catalogue no. ASR-001, Alomone Labs), SSTR2 control peptide (catalogue no. ab171899, Abcam), SSTR3 control peptide (catalogue no. ASR-003, Alomone Labs), peptide corresponding to the anti-SSTR4 antibody (amino acid sequence (C) QQEALQPEPGRKRIPLTRTTTF, Ontores, commercially not available), and SSTR5 control peptide (catalogue no. ASR-005, Alomone Labs).

The following primary antibodies were used to validate transgene specificity in transgenic F1 mice: rabbit anti-somatostatin polyclonal antibody (1:500, catalogue no. H-106, Santa Cruz), mouse anti-parvalbumin polyclonal antibody (1:1000, catalogue no. P3088, Sigma-Aldrich), and rabbit anti-Vasoactive Intestinal Peptide (1:500, catalogue no. 9535-0204, Bio-Rad). A donkey anti-rabbit IgG (H + L) Highly-Cross-Adsorbed, Alexa Fluor 488 (1:500, catalogue no. A-21206, Thermo Fisher Scientific), and donkey anti-mouse IgG (H + L) Highly-Cross-Adsorbed, Alexa Fluor 488 (1:500, catalogue no. A-21202, Thermo Fisher Scientific) were used as secondary antibodies.

### Immunohistochemistry

The mice were anaesthetised and transcardially perfused with 4% paraformaldehyde in 0.01 M phosphate-buffered saline (PBS). The brains were post-fixed for 24 h in 4% paraformaldehyde in PBS at 4 °C, cryoprotected in a series of sucrose solutions in PBS (10%, 20%, and 30%; 24 h each; 4 °C), cooled in n-heptane, placed on dry ice, and stored at − 80 °C. 30-μm sections were cut in a coronal and in a tangential plane through the barrel cortex using a cryostat (Leica CM1860 UV).

Free-floating sections were blocked for 20 min at room temperature in PBS containing 5% normal donkey serum (catalogue no. D9663, Sigma-Aldrich), 5% bovine serum albumin (catalogue no. A7906, Sigma-Aldrich), and 0.1% Triton-X (catalogue no. T8787, Sigma-Aldrich). Afterward, the sections were incubated overnight with the appropriate anti-SSTR primary antibody in blocking buffer at 4 °C. The next day, the samples were washed in PBS and incubated with a secondary antibody for 2 h at RT. Then, they were washed again in PBS, incubated for 2 min in 4′,6-diamidino-2-phenylindole (DAPI) solution and mounted using ProLong Glass Antifade Mountant (catalogue no. P36982, Thermo Fisher Scientific). The staining was performed separately for each SSTR using different transgenic (PV-Ai14, *n* = 4; SST-Ai14, *n* = 4; VIP-Ai14, *n* = 4) or wild type C57BL/6 (*n* = 4) mice.

### Image analysis

Immunofluorescent signals were analysed using confocal microscopy. Low-magnification coronal and tangential images of somatosensory cortex were obtained using a Zeiss Cell Observer SD Spinning Disk confocal microscope (10 × objective magnification) and Nikon ECLIPSE 80i microscope connected to Evolution VF digital camera (4 × objective magnification), while higher magnification images, used for more precise SSTRs localisation and quantitative analysis, were made with a Zeiss Laser Scanning confocal microscope LSM 800 with Airy Scan (10 ×, 40 ×, and 63 × objective magnification). The Allen Mouse Brain Atlas was used to identify the somatosensory cortex. The pictures were post-processed using the ImageJ program.

To semi-quantify the laminar distribution of SSTRs in the cortex, an intensity of immunofluorescence was measured using ImageJ software. Briefly, SSTRs fluorescence intensity profiles along the vertical column axis (pia to white matter across all cortical layers of barrel cortex) of the representative coronal brain slices were obtained with plot profile function of ImageJ.

To quantitatively assess the localisation of SSTRs in PV, SST and VIP INs, the percentage of each type of interneuron that was immunopositive for SSTR1-5 in the particular cortical layer (L2/3, L4 and L5/6) was defined using ImageJ cell counter plugins. Two coronal brain sections containing the barrel cortex from each transgenic mouse (PV-Ai14 *n* = 3, SST-Ai14 *n* = 3, VIP-Ai14 *n* = 3) were used for the analysis and 100% referred to all interneurons visible in the ROI. The borders between L1, L2/3, L4, and L5/6 were defined based on an anatomical atlas and DAPI staining. The obtained results were analysed using the GraphPad Prism 5 software (Inc.). Arithmetical mean ± SD was calculated for each group. Considering a low number of animals in each group (*n* = 3) non-parametric Kruskal–Wallis test was used, accepting significance border for *p* ≤ 0.05.

### Validation of antibodies and transgene specificity

**Fig. 1:**
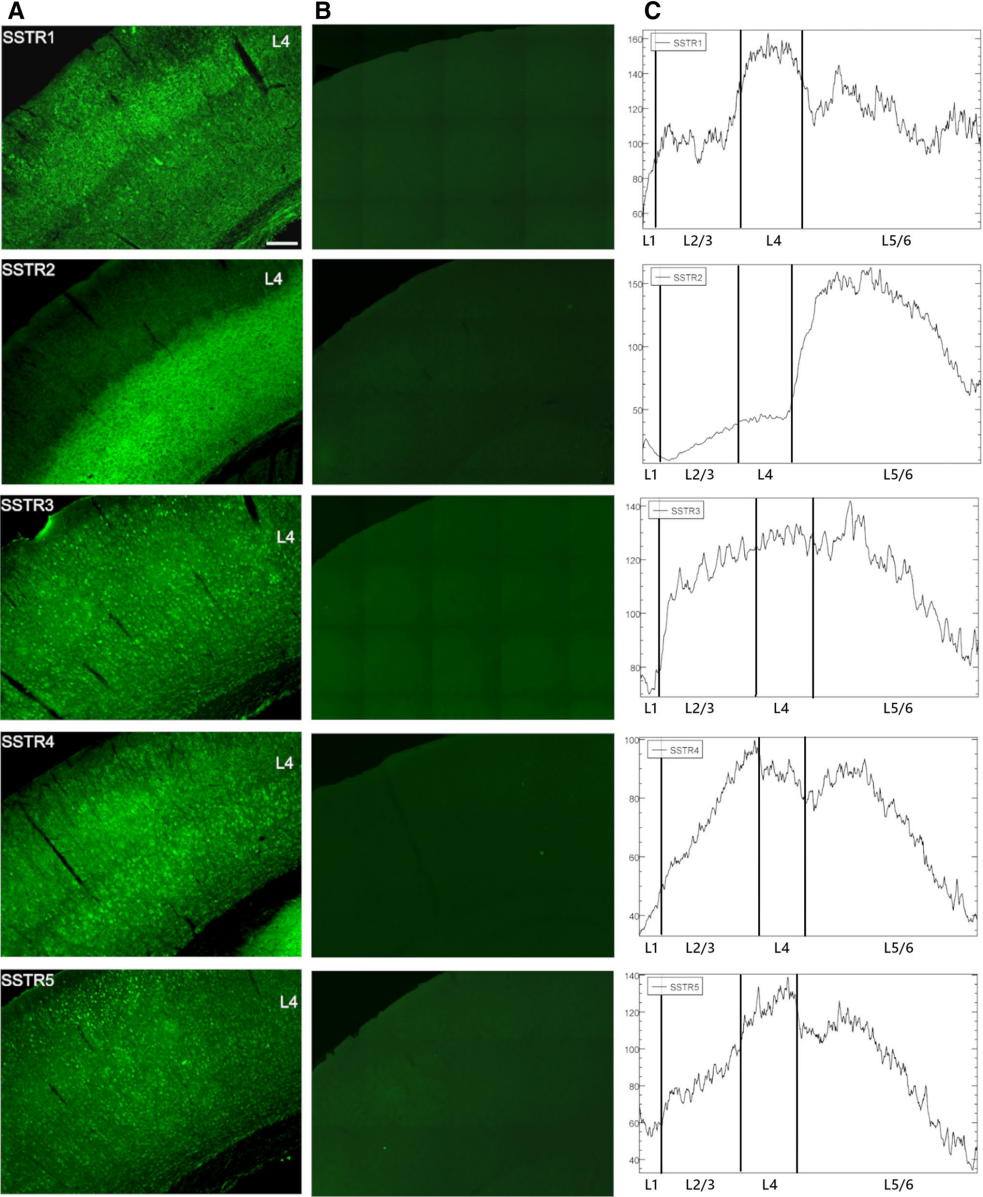
Localisation of SSTRs in the cortical layers of mice primary somatosensory cortex. SSTR1 showed the most intense signal in L4 and a weaker signal in L2/3 and L5/6. SSTR2 was localised mainly in deep cortical layers (L5-6). SSTR3, SSTR4, and SSTR5 were distributed evenly throughout the cortical layers (column A and C). **Column a** present representative coronal brain sections through somatosensory cortex immunofluorescently labelled with antibodies specific for SSTR1-SSTR5. **Column b** presents the validation of antibody specificity. Coronal brain sections were treated with primary antibodies (anti-SSTR1-5) preincubated with control blocking peptides, followed by specific secondary antibodies. The addition of control peptides resulted in the disappearance of the antibody-specific signal. Scale bar 100 μm

To validate the specificity of anti-SSTR antibodies, we performed experiments using blocking peptides corresponding to the antibodies that were used. The primary antibodies were incubated with suitable peptides at a 1:10 ratio for 3 h at RT prior to incubation with brain sections. Other steps were performed as described in the *Immunohistochemistry* section*.* For these experiments, wild type C57BL/6 mice (*n* = 3) were used. The results confirmed the specificity of the antibodies used in the experiments (Fig. 1b).

To confirm the specificity of transgene expression in the PV-Ai14, SST-Ai14, and VIP-Ai14 mice, we performed a series of immunostaining using brain samples from the transgenic mouse lines in combination with anti-PV, anti-SST, and anti-VIP antibodies. We have shown that the fluorescence signal expressed by the transgene overlaps with the fluorescence signal of the antibodies (not shown). The distributions of the particular interneuron types in the transgenic lines were similar to those described previously in the mouse sensory cortex (Gentet et al. 2012; Neske et al. 2015).

## Results

### SSTR distribution in cortical layers

Each SSTR was present in all cortical layers of the mouse somatosensory cortex, presenting a specific distribution. SSTR1 showed the most intense signal in L4 and weaker signal in L2/3 and L5/6. SSTR2 was localised mainly in the deep cortical layers (L5–6). The SSTR3 and SSTR5 were distributed more evenly among all of the cortical layers. The immunoreactivity of SSTR4 was stronger in L2/3 and L5 compared to other cortical layers (Fig. 1a). Those observations were confirmed semi-quantitatively with immunofluorescence intensity profiles along the vertical column across the barrel cortex (Fig. 1c).

**Fig. 2:**
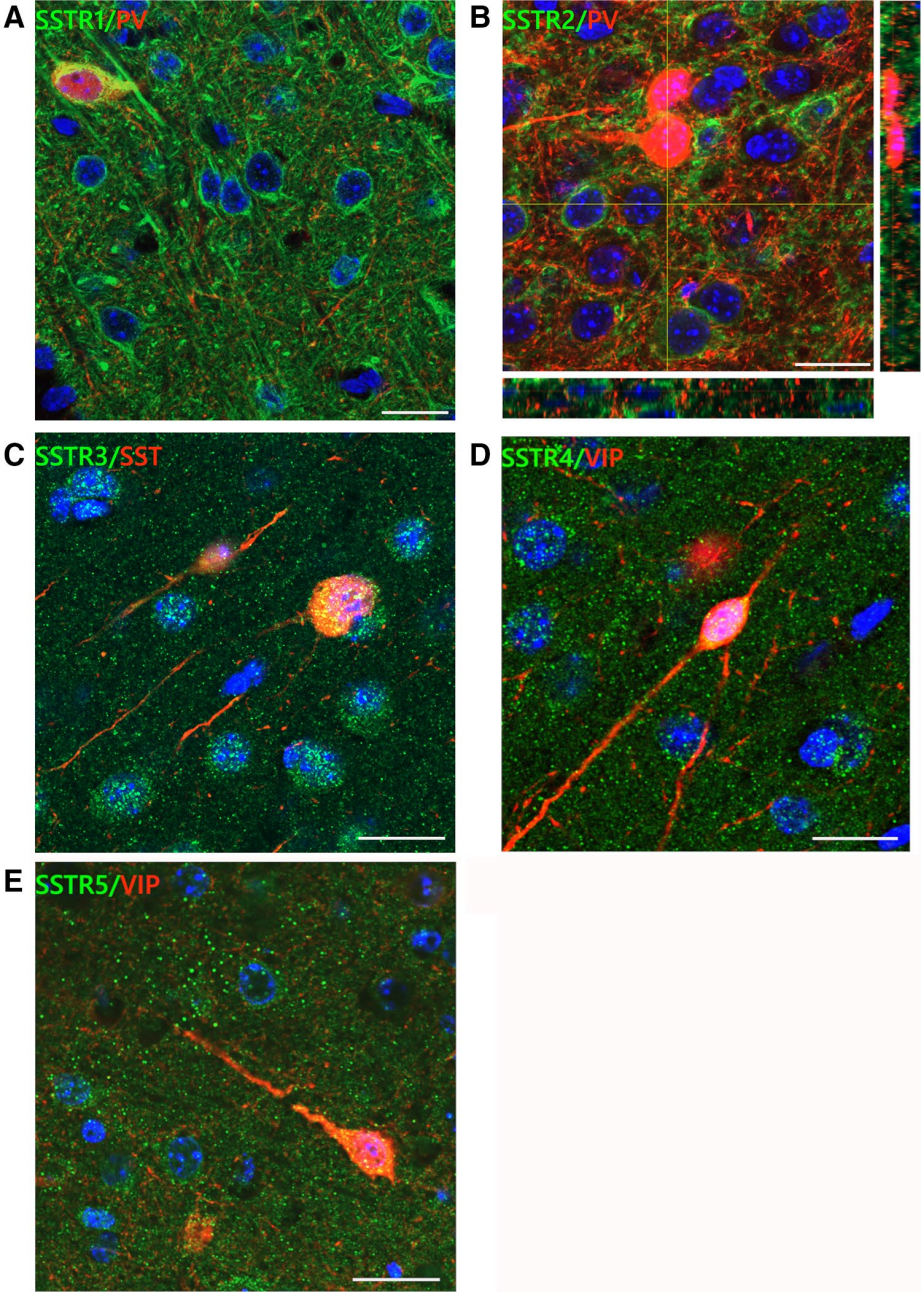
SSTRs localisation in the somatosensory cortex of mice. High magnification confocal pictures of coronal sections presenting localisation of SSTR1-5 in the mouse somatosensory cortex. **a** SSTR1 labelled clearly the pyramidal-like cell bodies and apical dendrites. **b** Lack of co-localisation of SSTR2s and PV INs in the mouse primary somatosensory cortex. Yellow lines represent two orthogonal sections of a z-series showing the distribution of green and red fluorescence in the tissue. **c** SSTR3 immunoreactivity was found in cell bodies and neuropil. **d, e** Immunofluorescent signal for SSTR4 and SSTR5 was found mainly in the neuropil and much less visible in cell bodies. Scale bar for all pictures: 10 μm

SSTRs were present both in cells and the neuropil with immunoreaction detected both at the plasma membrane and in the cytoplasm. However, anti-SSTR1 and anti-SSTR2 antibodies labelled neuronal cell bodies and processes very intensely, while SSTR3-5 exhibited weaker labelling intensity and seemed to be localised mainly in the soma with dispersed neuropil staining. Particularly, strong immunolabelling of pyramidal cell bodies and apical dendrites was obtained with the anti-SSTR1 antibody in L2/3 and L5 (Fig. 2; supplementary figures).

### SSTRs distribution pattern in the barrel cortex

**Fig. 3:**
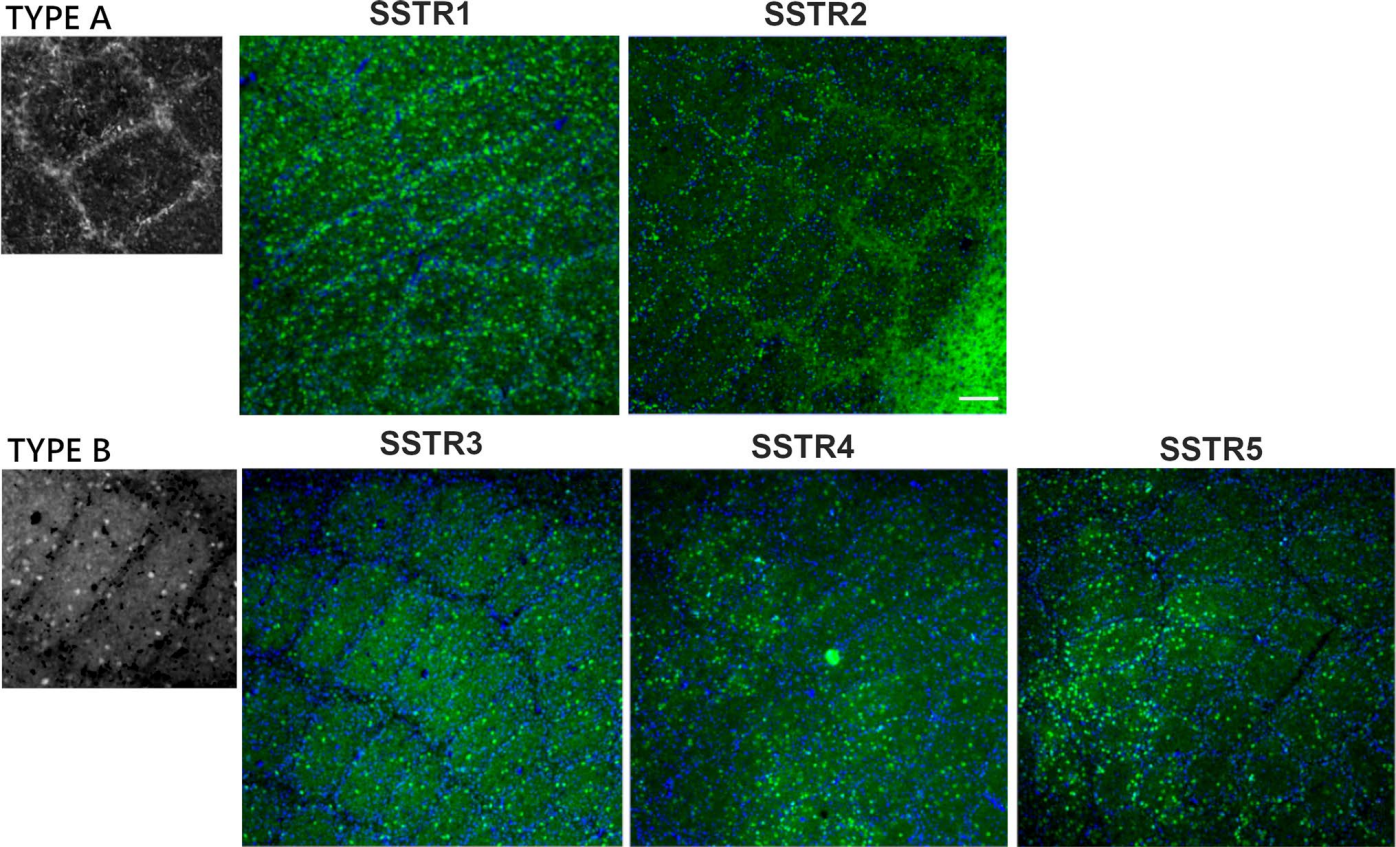
Two types of SSTRs distribution in the mouse barrel field. Tangential brain sections through L4 of the mouse barrel cortex. Type A distribution is characterised by a high concentration of immunoreactivity in the barrel walls (sides + septa). This type is represented by SSTR1 and SSTR2. Type B distribution is characterised by a homogeneous intensity of immunoreactivity in the barrel hollows. This type is represented by SSTR3, SSTR4, and SSTR5. Scale bar: 100 μm. Nuclear DAPI staining is visible in blue

Depending on the specific pattern of the SSTRs localisation in the mouse barrel cortex, we classified them into two types. The first one (type A) was characterised by a high receptor concentration in the barrel walls, while the second type (type B) was characterised by a homogeneous receptor distribution in the barrel hollows. Type A distribution pattern was represented by SSTR1 and SSTR2, while SSTR3, SSTR4, and SSTR5 belonged to type B (Fig. 3). In the case of SSTR2, this pattern was most visible at the border of layers 5 and 4.

### SSTRs localisation in PV, SST, and VIP interneurons

The densities of particular interneurons containing individual SSTRs in the different layers of the cerebral cortex are presented in supplementary Table S1. The data represent the mean value of cell number per area [mm^2^] ± standard deviation. Additionally, the percentages of double immunopositive cells (PV-/SST-/VIP- and SSTRs-positive cells) are listed in the table**.**

### PV INs

**Fig. 4:**
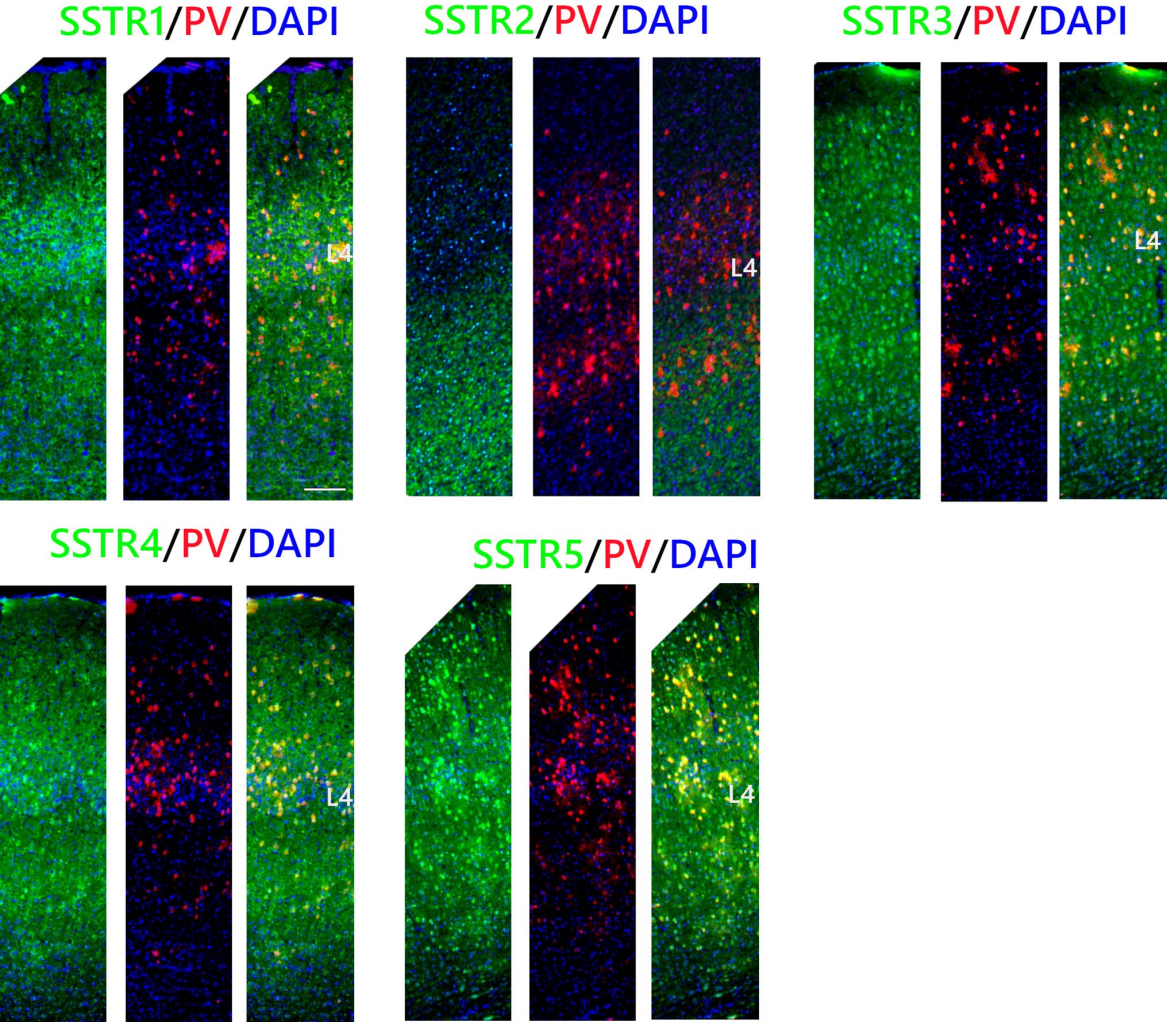
Co-localisation of SSTRs with PV containing interneurons in the mouse primary somatosensory cortex. Coronal sections demonstrating the co-localisation of particular SSTRs (green immunofluorescence) with PV containing interneurons (red tdTomato fluorescence); DAPI staining shows the nuclei (blue). Scale bar: 100 μm

Observations using high objective magnification revealed that SSTR1 was present on 85 ± 9% of PV INs in L2/3 and on 77 ± 7% of PV INs in L4 and L5/6. SSTR3 and SSTR5 presented very similar patterns of co-localisation and the majority of PV INs were positive for both receptors in all cortical layers. The percentage of double-positive cells was as follows: L2/3: 74 ± 10% for SSTR3 and 74 ± 5% for SSTR5; L4: 90 ± 6% for SSTR3 and 75 ± 7% for SSTR5; L5/6: 96 ± 2% for SSTR3 and 93 ± 7% for SSTR5. SSTR4 was most abundant in the PV cells in L4, reaching 62 ± 14%. In layer 2/3, 40 ± 12% of the PV cells were SSTR4-positive and in L5/6, co-localisation was observed in 36 ± 7% of the interneurons (Figs. 4, 7).

Importantly, we did not observe the localisation of SSTR2 in PV INs in any layer (Figs. 2, 4, 7).

### SST INs

**Fig. 5:**
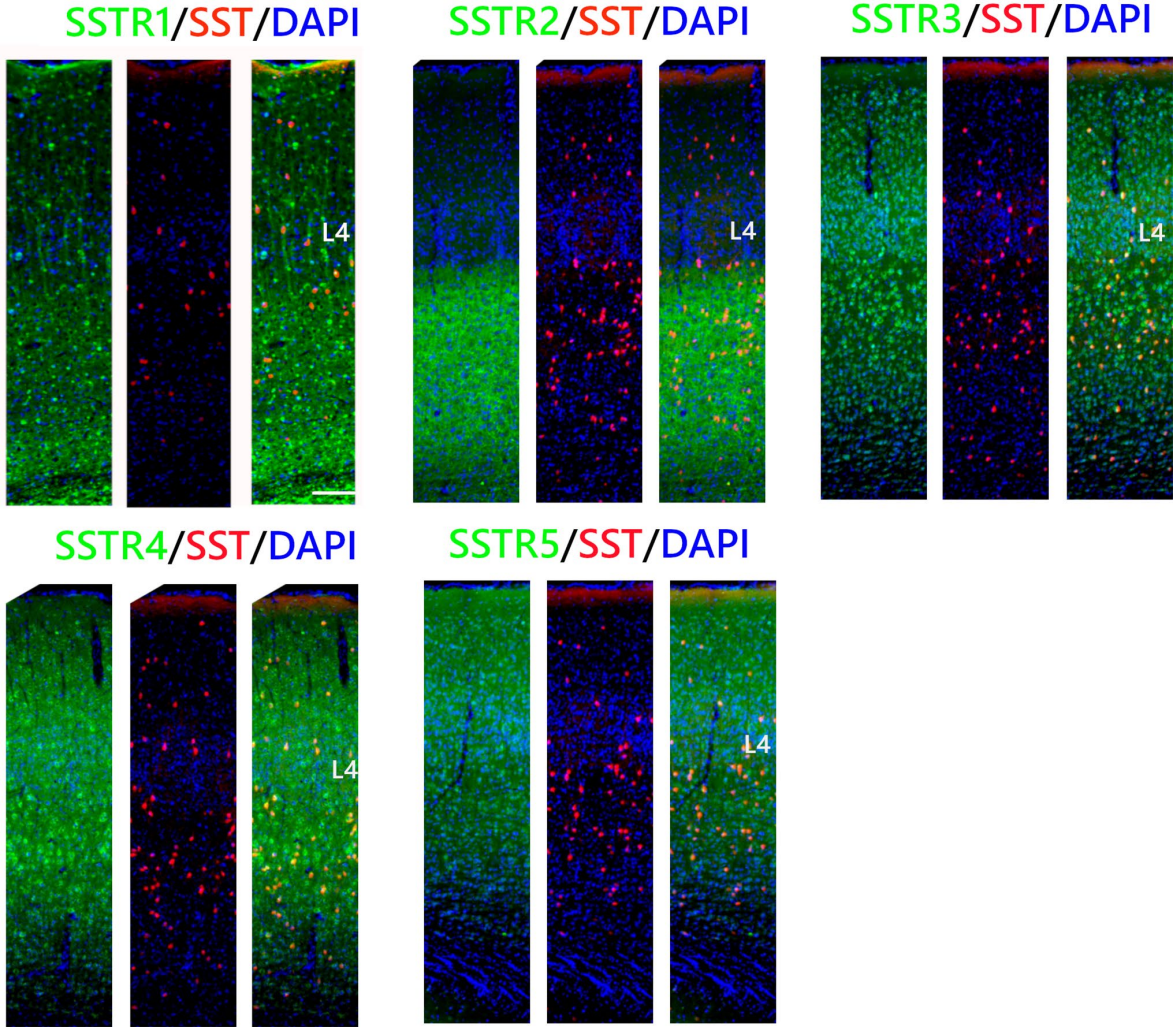
Co-localisation of SSTRs with SST containing interneurons in the mouse primary somatosensory cortex. Coronal sections demonstrating the co-localisation of particular SSTRs (green immunofluorescence) with SST containing interneurons (red tdTomato fluorescence); DAPI staining shows the nuclei (blue). Scale bar 100 μm

Most of the SST cells in the somatosensory cortex were SSTR1-, SSTR3-, and SSTR5-positive, especially in L5/6. Percentage analysis indicated that 66 ± 13% of the SST INs in L2/3, 74 ± 11% in L4, and 93 ± 4% in L5/6 expressed SSTR1. In the case of SSTR3, 81 ± 7% of the SST cells in L2/3, 91 ± 5% in L4, and up to 97 ± 2% in L5/6 were double labeled. The expression of SSTR5 was observed in 90 ± 8% of the SST cells in L2/3 and L5/6 and 83 ± 5% of the SST cells in L4. Visibly fewer SST INs showed immunoreactivity for SSTR4: 66 ± 10% of the SST INs in L2/3, 61 ± 8% in L4, and 49 ± 12% in L5/6 and for SSTR2: 36 ± 9% of the SST INs in L2/3, 35 ± 6% in L4, and only 17% in L5/6 (Figs. 5, 7).

### VIP INs

**Fig. 6:**
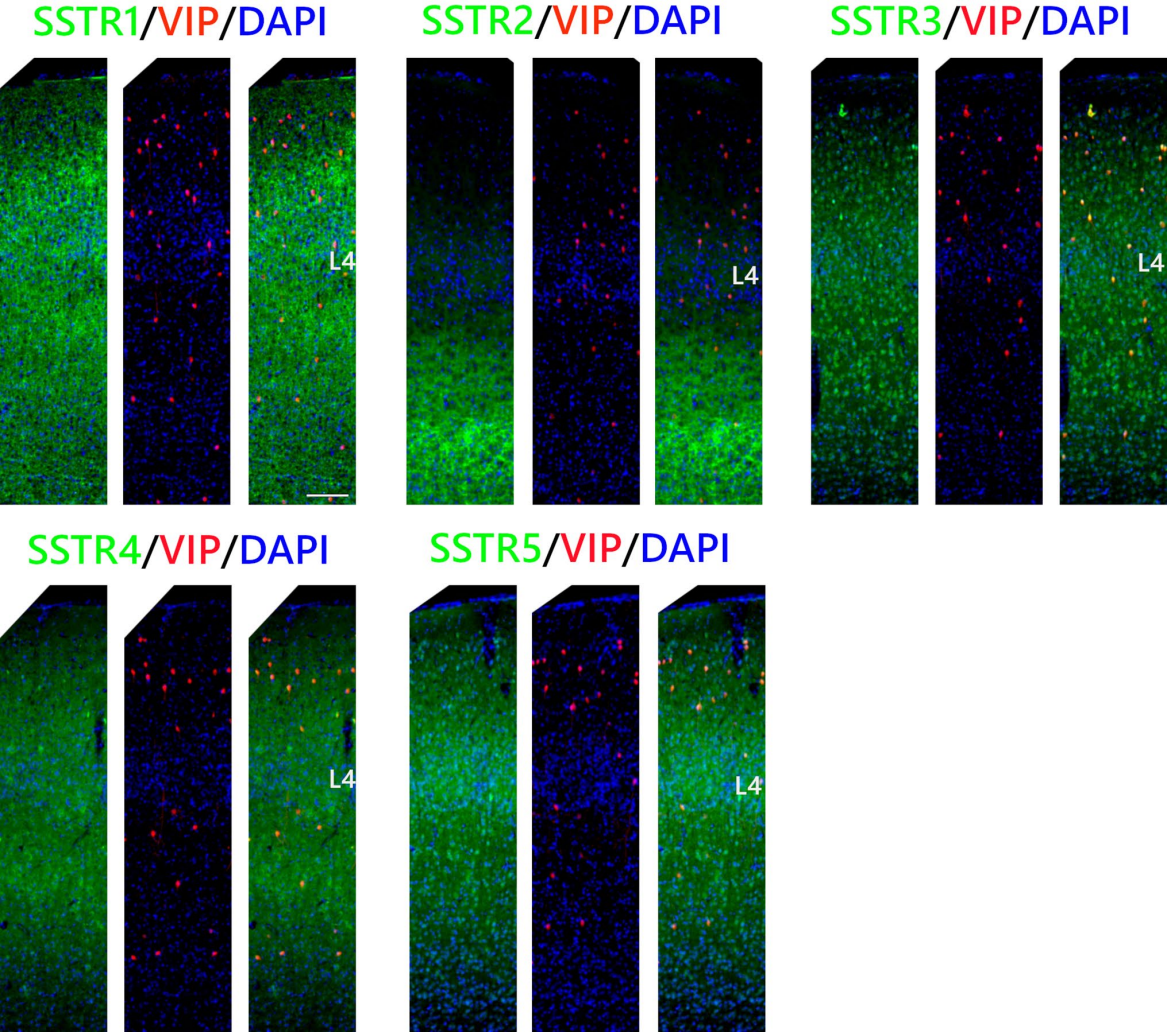
Co-localisation of SSTRs with VIP containing interneurons in the mouse primary somatosensory cortex. Coronal sections demonstrating the co-localisation of particular SSTRs (green immunofluorescence) with VIP containing interneurons (red tdTomato fluorescence); DAPI staining shows the nuclei (blue). Scale bar 100 μm

**Fig. 7:**
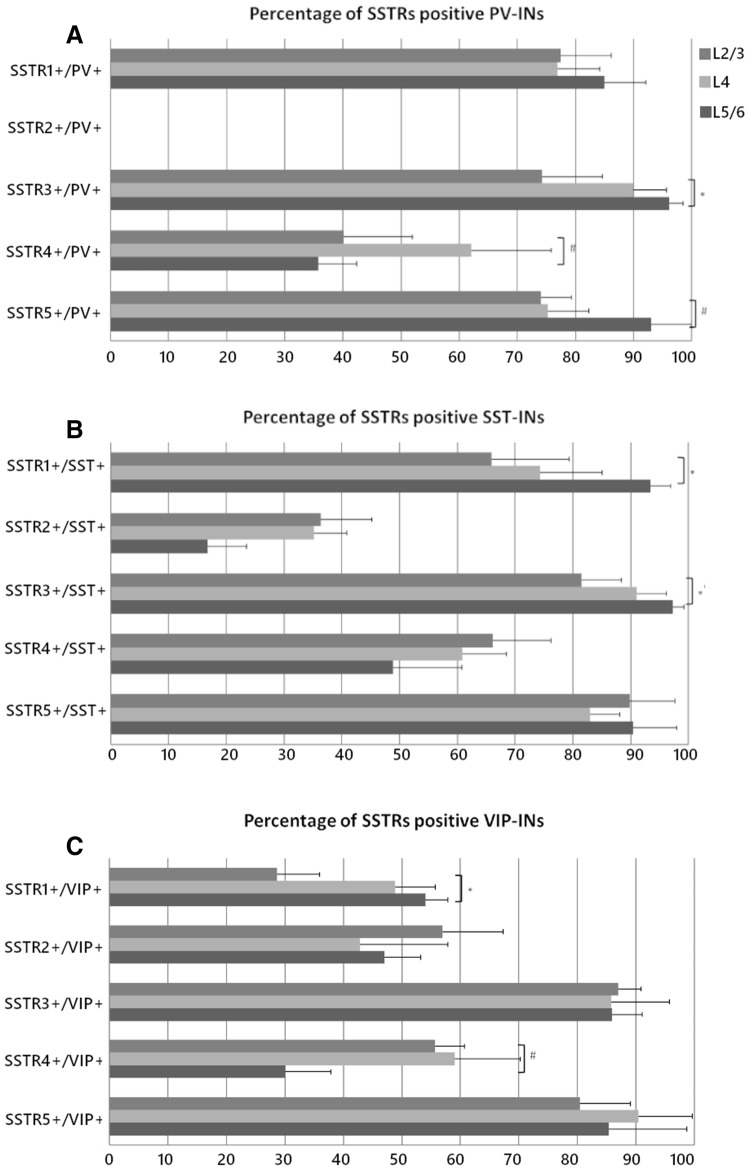
Quantitative assessment of co-localisation of SSTRs with PV, SST, and VIP containing INs in the particular cortical layers of the mouse somatosensory cortex. Graphs represent the percentage (mean + SD) of all labelled interneurons of each particular type that exhibited co-localisation with SSTRs in different cortical layers. Top panel: SSTR1-5 + PV INs, Middle panel: SSTR1-5 + SST INs, Bottom panel: SSTR1-5 + VIP INs. Kruskal–Wallis test, *p ≤ 0.05, ^#^0.05 < *p* < 0.051; *n* = 3

In the case of VIP INs, the percentages of the cells that were double immunopositive for SSTR3, SSTR4, or SSTR5 and VIP were similar to those found in SST INs: 87 ± 4% in L2/3, 86 ± 10% in L4, and L5/6 86 ± 5% for SSTR3; 30 ± 8% in L2/3, 59 ± 11% in L4, and 56 ± 5% in L5/6 for SSTR4 and 80 ± 9% in L2/3, 90 ± 9% in L4, and 85 ± 13% in L5/6 for SSTR5). However, among VIP INs there was a significantly lower percentage of neurons that expressed SSTR1 than in any other analysed INs types. The expression of SSTR1 was observed in 29 ± 7% of the VIP INs in L2/3, 49 ± 7% in L4, and 54 ± 4% in L5/6. Finally, the percentages of the cells expressing SSTR2 were 57 ± 10% in L2/3, 43 ± 15% in L4, and 47 ± 6% in L5/6 (Figs. 6, 7).

## Discussion

We found that SST interneurons and VIP interneurons expressed all five SSTR subtypes, with some laminar specificity. Interestingly, PV interneurons did not express SSTR2, one of the predominant somatostatin receptors in the cerebral cortex (Bologna and Leroux 2000). The presence of all five SSTRs has been previously demonstrated in the cortex (reviewed in Csaba and Dournaud 2001) and our results nicely complement those earlier observations, as we have shown that particular SSTRs have a specific distribution within the barrel cortex of mice. It was shown that SSTR1 and SSTR2 are two of the most predominant SSTRs in the human and rat cerebral cortices (Bologna and Leroux 2000; Kumar 2005). We observed much higher intensity of SSTR1 than SSTR2 signal in the superficial layers, while SSTR3-5 presented more even distribution throughout the cortex. Low levels of SSTR2 in upper cortical layers were earlier described both by in situ hybridisation experiments (Gonzalez et al. 1991; Reubi et al. 1986; Perez et al. 1994; Señarís et al. 1994; Beneyto et al. 2012) and by immunocytochemistry (Kumar 2005; Schindler et al. 1996; Adori et al. 2015). It appears that the well-recognised inhibition of apical dendrites of pyramidal neurons by SST INs is exerted rather by SSTR1, which shows high immunoreactivity in the upper cortical layers.

Interestingly, a detailed analysis of SSTRs distribution in the barrel cortex revealed two distinct patterns: types A and B. Type A was represented by SSTR1 and SSTR2, which showed a dense distribution in the barrel walls (sides and septa) resembling so-called “barrel nests”, a pattern of L2/3 axons running preferentially in the septal regions of layer 4 (Sehara et al. 2010). On the other hand, type B, represented by SSTR3-5, was distributed within the barrel hollows. The anatomical and physiological evidences indicate that the barrel-related and septa-related circuits represent two processing streams that differ in their response patterns and computational functions (Alloway 2008). Barrel-related circuitry is related to the sensory processing of spatiotemporal information associated with tactile stimuli, while septal-related circuitry encodes the kinetics of whisker movements and controls whisker motor behaviour (Alloway 2008). The barrel and septal circuits must work together to discriminate objects detected by passive or active whisker movements, but the exact neural mechanisms of this cooperative process are not known. Barrel nests could mediate the integration of these two separate circuits and the segregation of SSTRs into two types of distributions corresponding to the two information streams in the barrel cortex may suggest a different involvement of SSTR of each type in information processing in those two pathways.

We found that almost all SSTRs co-localised with the three examined populations of inhibitory interneurons in the somatosensory barrel cortex; however, the percentage of interneurons containing each SSTR depended on the interneuron type and the cortical layer. SSTR3 and SSTR5 were similarly strongly co-localised with all types of interneurons and this co-localisation ranged from 74 to 97%. Some authors have shown that in mouse and rat, SSTR3 is selectively targeted to primary neuronal cilia, a specialised microtubule-based organelle that play a role in neuronal signaling and the regulation of synaptic connectivity (Handel et al. 1999; Whitfield 2004; Guo et al. 2017). We detected SSTR3 in membrane and cytoplasm of neuronal cell bodies and dendrites, while observing the cilia-like shapes as well. Such a pattern of SSTR3 distribution in cilia, but also in cell bodies was reported by Green and Mykytyn 2010 and Guadiana et al. 2016. SSTR5 is involved in the regulation of cAMP/PKA/ERK1/2 signaling pathways, which influence gene expression, cell division, differentiation, and apoptosis (Zou et al. 2017). Moreover, SSTR5 acting postsynaptically inhibits adenylate cyclase and induces inwardly rectifying potassium channel leading to cell hyperpolarisation, which closes voltage-dependent calcium channels, inhibiting the neuron (Moller et al. 2003; Alexander et al. 2017; Zou et al. 2017). Thus, the presence of SSTR5 on PV, SST, and VIP INs suggests that these receptors may be involved in the phenomenon of disinhibition.

The SSTR1 was expressed on PV and SST INs more frequently than on VIP INs. Although VIP INs are 5HT3aR-expressing cells (Lee et al. 2010; Tremblay et al. 2016), they can exhibit different morphological (bipolar or multipolar) and electrophysiological properties (Tremblay et al. 2016). Presumably, the presence of SSTR1 only on a certain fraction of VIP INs (29–54%) may be one more factor distinguishing a particular subpopulation of VIP INs in the primary somatosensory cortex. Considering that SSTR1 is mainly presynaptic (Schulz et al. 2000) and that its activation results in the inhibition of GABAergic transmission in presynaptic neurons (Leresche et al. 2000), we can assume that SSTR1 may weaken VIP-to-SST signaling, thereby strengthening the effect of SST on PCs. This effect would oppose the classic pathway in which VIP INs create preferential connections with SST INs and inhibit them, which results in the disinhibition of pyramidal excitatory neurons (Tremblay et al. 2016; Yetman et al. 2019; Zhang et al. 2016).

Perhaps the most interesting result concerns the lack of the localisation of SSTR2 on the cell bodies and proximal dendrites of PV INs regardless of the cortical layer. All other examined INs were SSTR2-immunopositive*.* It was demonstrated that the predominant localisation of SSTR2 in the rat cortex is on cell bodies and dendrites of pyramidal neurons (Schindler et al. 1996). Kumar (2005) observed that SSTR2 has low expression in non-pyramidal neurons. However, we observed SSTR2 on VIP INs (43–57%) and SST INs (17–36%). Therefore, it seems that PV interneurons may be regulated by SST INs via different mechanisms than other types of interneurons or PCs. SSTR2 is the only SST receptor that has two isoforms, SSTR2A and SSTR2B, in humans and rodents, and it was shown to transduce signals in a protein G-independent mechanism via interaction with beta-arrestin, which is followed by receptor internalisation (Grant et al. 2008). Strong predominance of SSTR3 and SSTR5 on interneurons suggests that these two receptors are most heavily involved in the somatostatinergic regulation of INs in the somatosensory cortex.

In the case of SST INs, we observed a significantly higher percentage of co-localisation with SSTR4 in L4 than in other layers. It was shown that SST INs and SSTR4 play regulatory roles in anxiety and mild stress-induced responses in the amygdala (Li et al. 2013; Scheich et al. 2016, 2017). Thus, it can be supposed that, in the barrel cortex, SSTR4 may be involved in fear-induced plastic changes, as our research has indicated the involvement of SST INs in this type of plasticity (Cybulska-Klosowicz et al. 2013).

An unexpected observation was the presence of all SSTR subtypes on SST INs. Several electrophysiological and anatomical papers have reported that SST interneurons, as opposed to PV INs, do not make connections with each other via chemical synapses. Simultaneous whole-cell recordings from labelled PV, VIP, and SST interneurons in L2/3 of the somatosensory cortex have shown that synaptic inhibition is completely absent among SST INs (Karnani et al. 2016b). Moreover, Pfeffer et al. (2013), using photostimulation of SST-Cre-ChR2 expressing neurons, have shown that SST INs, in contrast to PV INs, inhibit other subpopulations of interneurons rather than one another. However, SST INs were found to co-localise with SSTR1, 3 and 4 in the rat hypothalamus (Helboe et al. 1998; Kumar 2007), where they autoregulate their own secretion (Peterfreund and Vale 1984; Richardson and Twente 1986). Moreover, in the hypothalamus, SSTR1 and SSTR2 mRNAs were expressed in neurons containing somatostatin. It has been suggested that these two receptors can act as autoreceptors (Beaudet et al. 1995). A detailed analysis of the concomitance of SST and SSTR2A in the rat brain revealed that, within regions of somatodendritic labelling, a subpopulation of SSTR2A-immunoreactive cells was also immunopositive for SST in many brain structures, including the cortex, suggesting that a subset of SST2A receptors were autoreceptors (Dournaud et al. 1998). Thus, it may be assumed that the presence of SSTR1-5 on SST INs can be also related to autoregulation in the mouse cortex. Another possibility is that SSTRs are located extrasynaptically and act via volume transmission. Extrasynaptic receptors can be powerful regulators of neuronal activity and in the striatum somatostatin was shown to modulate the activity of neurons and glia via extrasynaptic receptors (Fuxe et al. 2012). Additionally, according to the EM study of SSTR2a distribution (Dournaud et al. 1998), a large fraction of SSTR2 is located intracellularly, this may be true also for other SSTRs.

Among the three subgroups of cortical interneurons analysed in the present manuscript, SST INs are probably the most heterogeneous group, regarding morphology and electrophysiological characteristics. The literature describing new somatostatinergic circuits is rapidly expanding (Naka et al. 2019). Therefore, detailed characteristics of the cortical distribution patterns of the SSTR subtypes on SST, PV and VIP INs may be a useful tool for further GABAergic interneuron classification. As each SSTR is a product of different gene, the expression of the different SSTRs can also complement the molecular characterisation of inhibitory interneurons.

## Electronic supplementary material

Below is the link to the electronic supplementary material.

Supplementary file1 (PDF 21297 kb)
