# Supplementary material for: Somatostatin receptors (SSTR1-5) on inhibitory interneurons in the barrel cortex
Source: Brain Struct Funct. 2019 Dec 23;225(1):387–401. doi: 10.1007/s00429-019-02011-7 (PMC6957562; doi:10.1007/s00429-019-02011-7)
Supplement: Supplementary file 1 — Supplementary file1 (PDF 21297 kb) [file 429_2019_2011_MOESM1_ESM.pdf]

Supplementary figures

Figures present colocalization of particular SSTRs (SSTR1-SSTR5) with three analysed types of interneurons (PV, SST and VIP) in different layers of mouse somatosensory cortex.
Each panel consists of confocal microscopy images of representative coronal sections. First column shows SSTRs in green + DAPI in blue; Second column shows INs in red + DAPI in blue; Third column presents colocalizations. Scale bars: 10 µm.

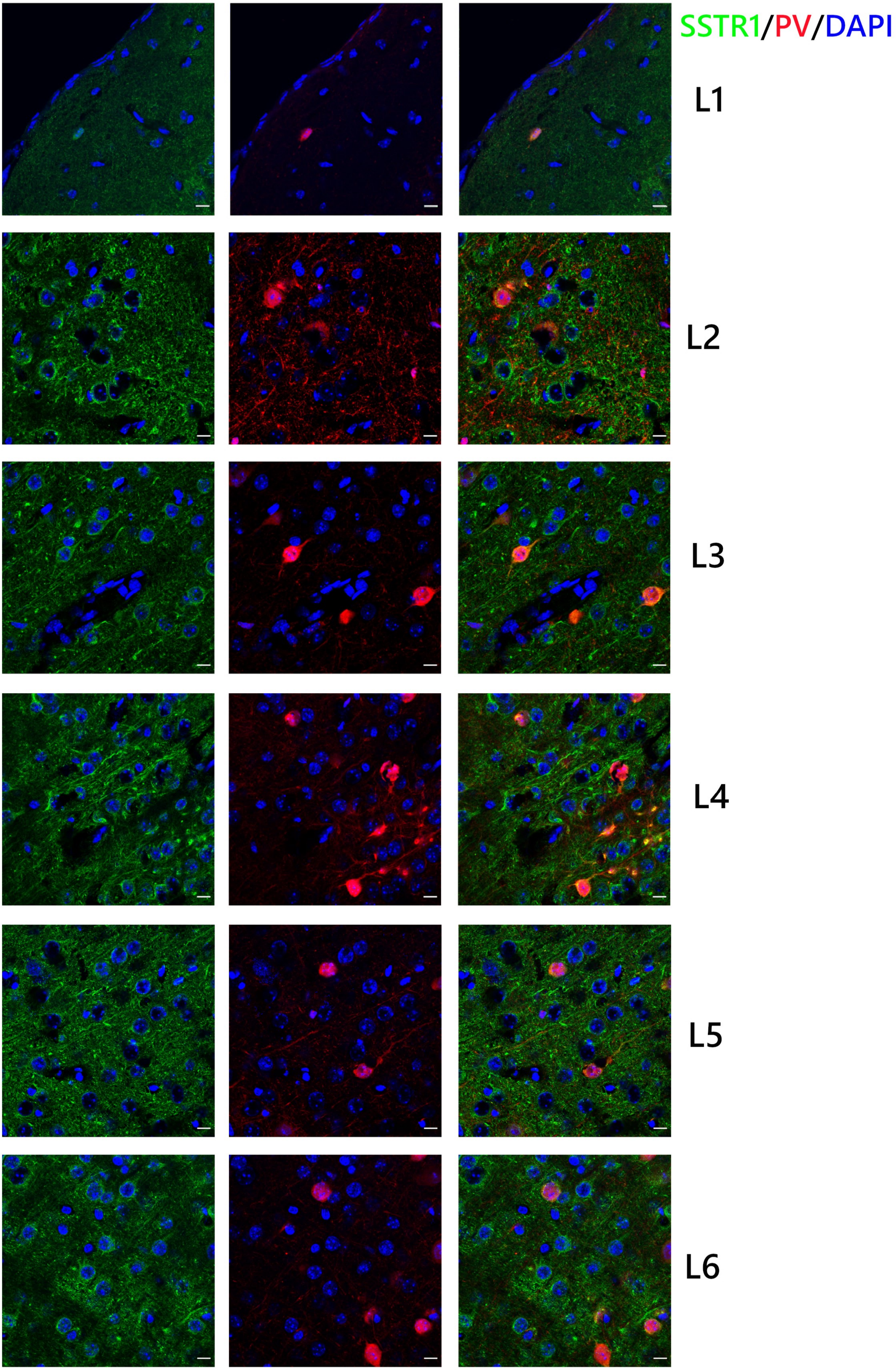

SSTR1/PV/DAPI
L1
L2
L3
L4
L5
L6

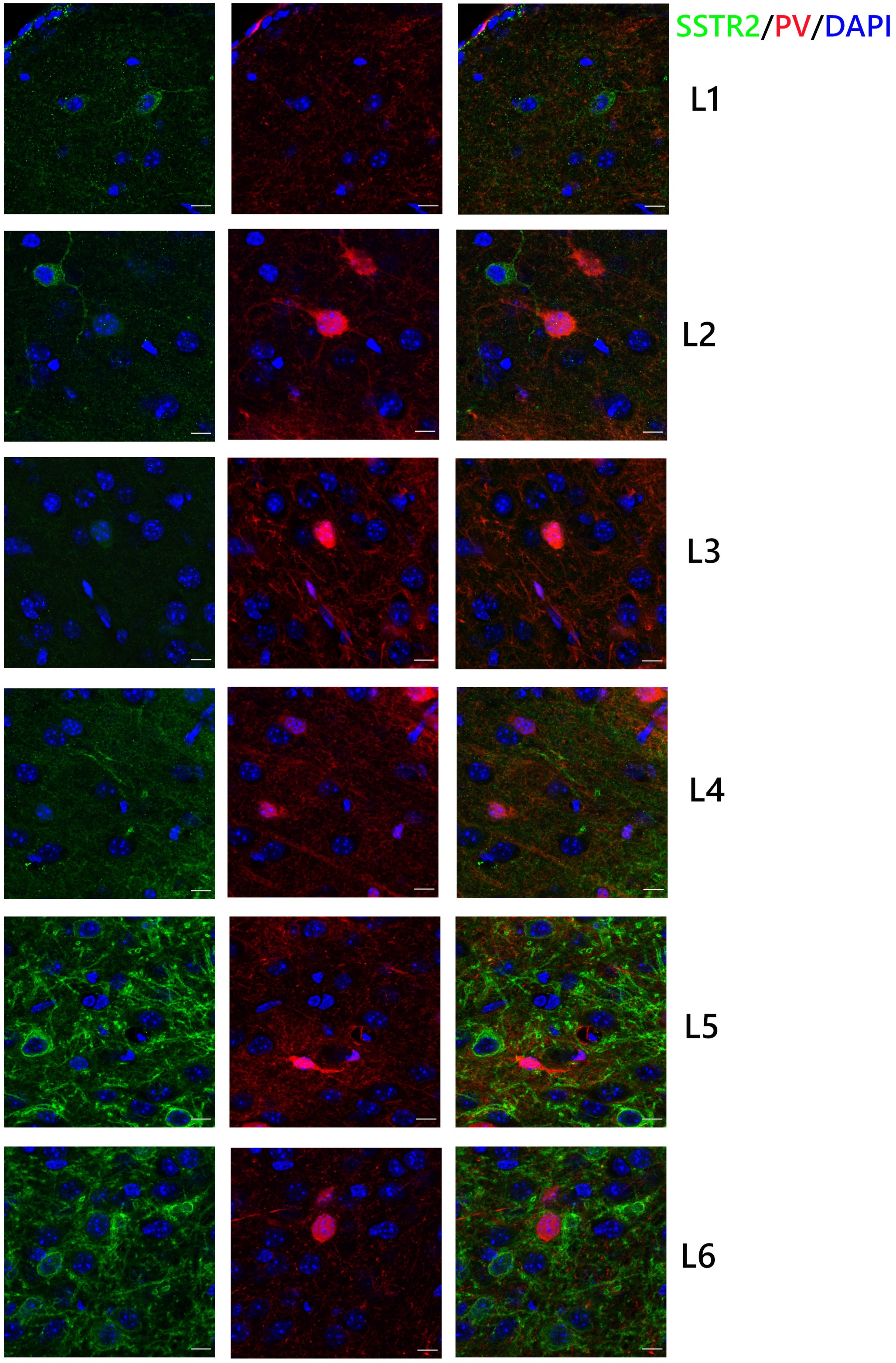
SSTR2/PV/DAPI
L1
L2
L3
L4
L5
L6

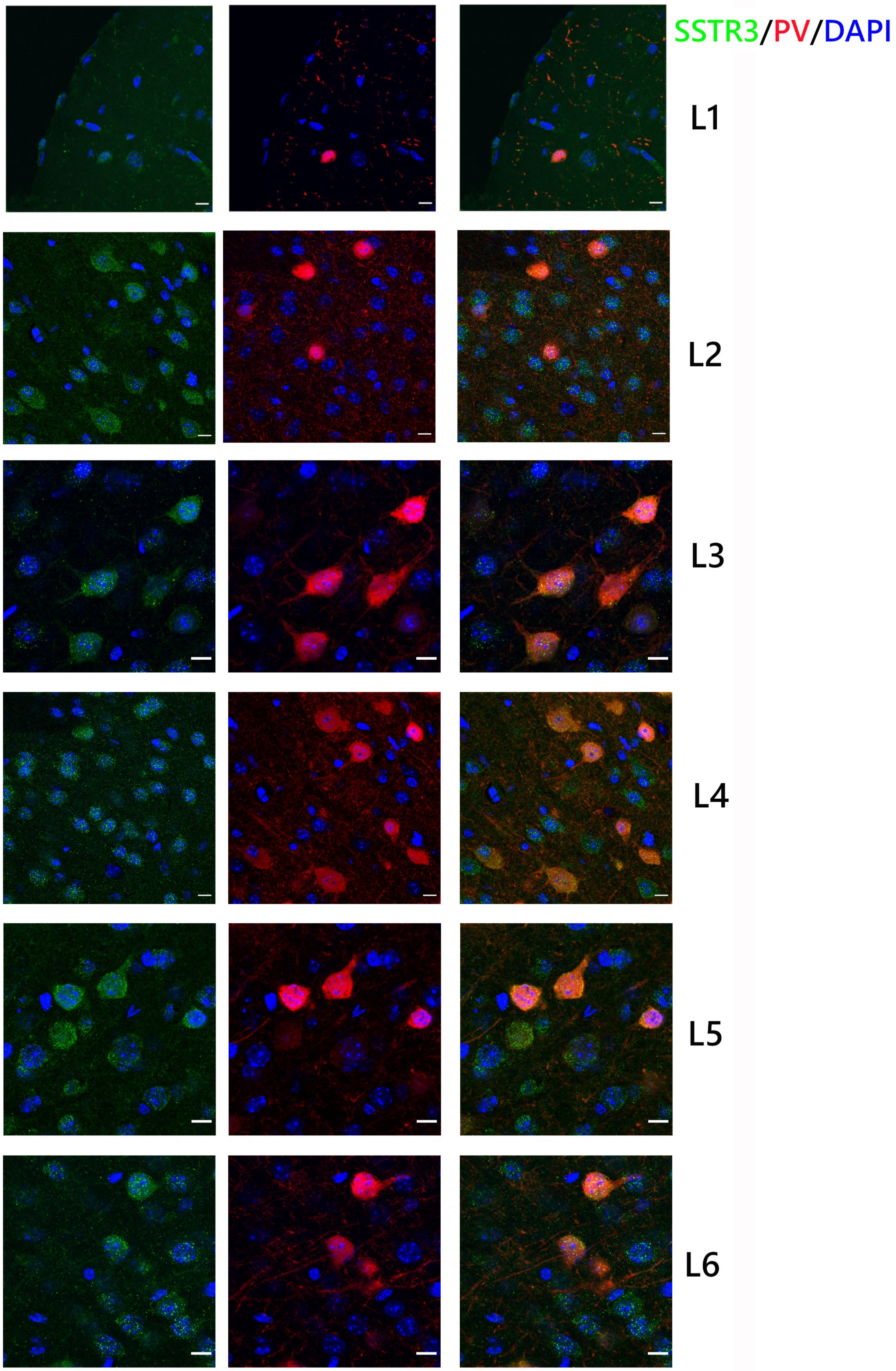
SSTR3/PV/DAPI
L1
L2
L3
L4
L5
L6

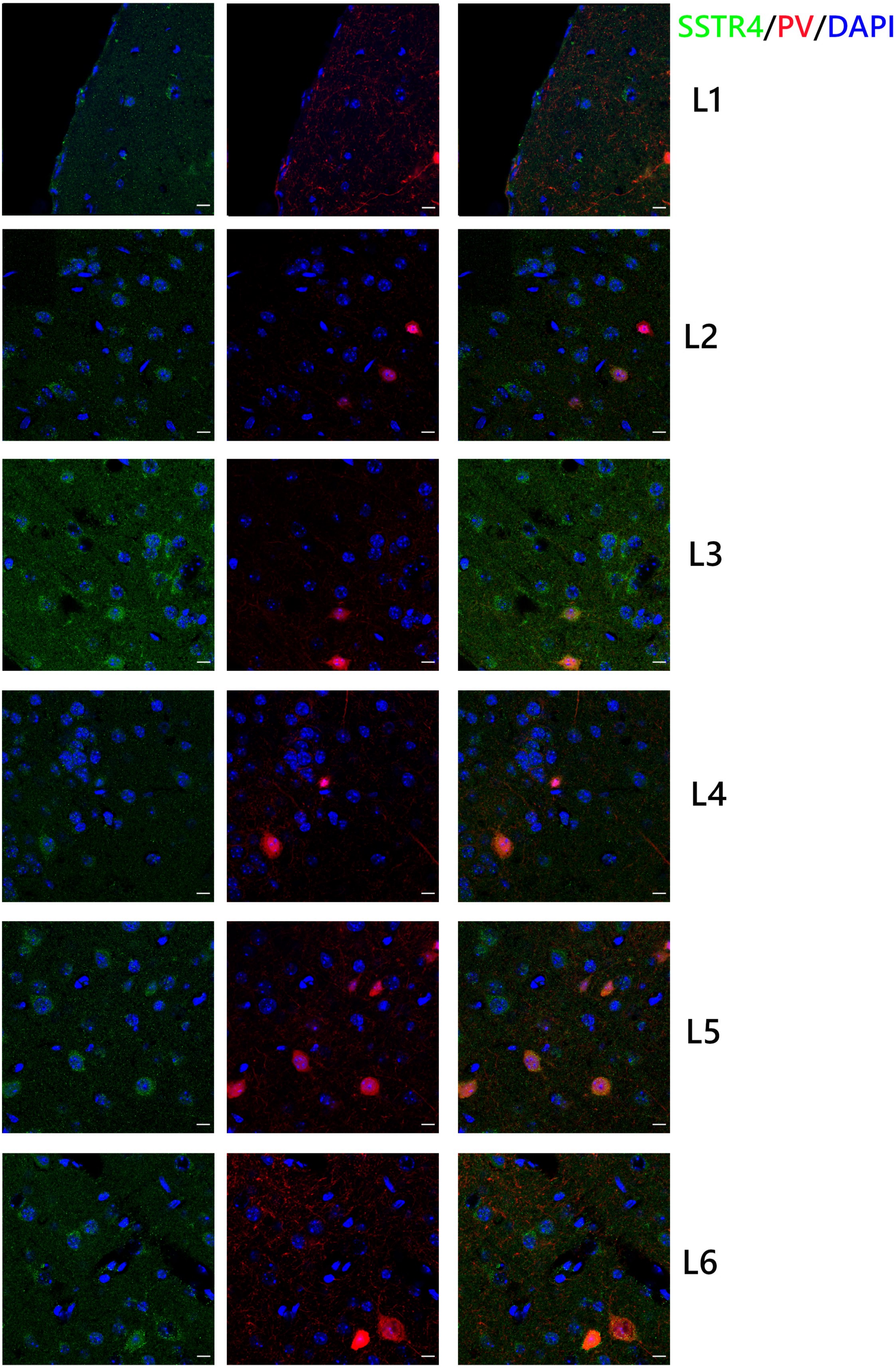
SSTR4/PV/DAPI
L1
L2
L3
L4
L5
L6

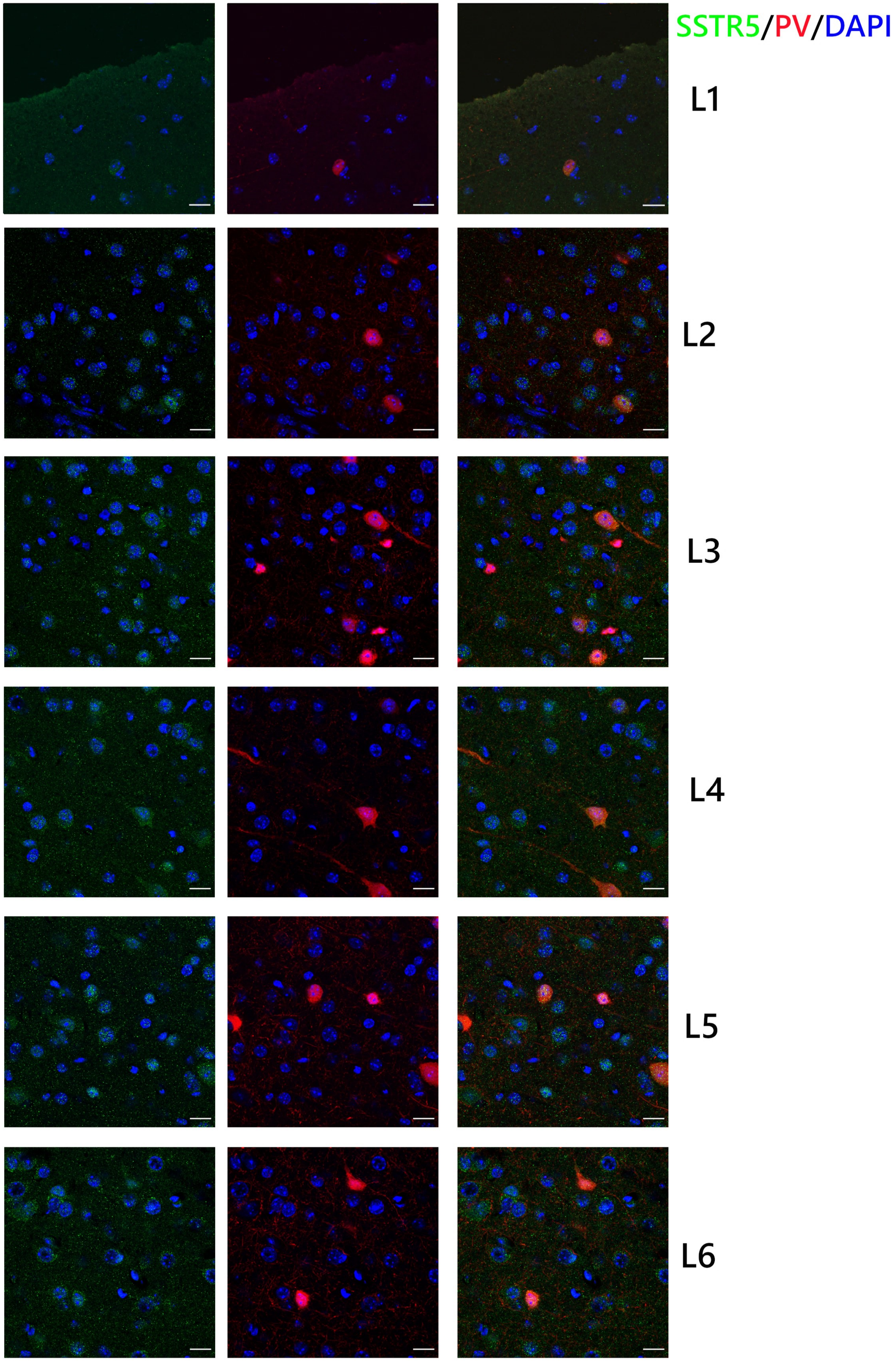
SSTR5/PV/DAPI
L1
L2
L3
L4
L5
L6

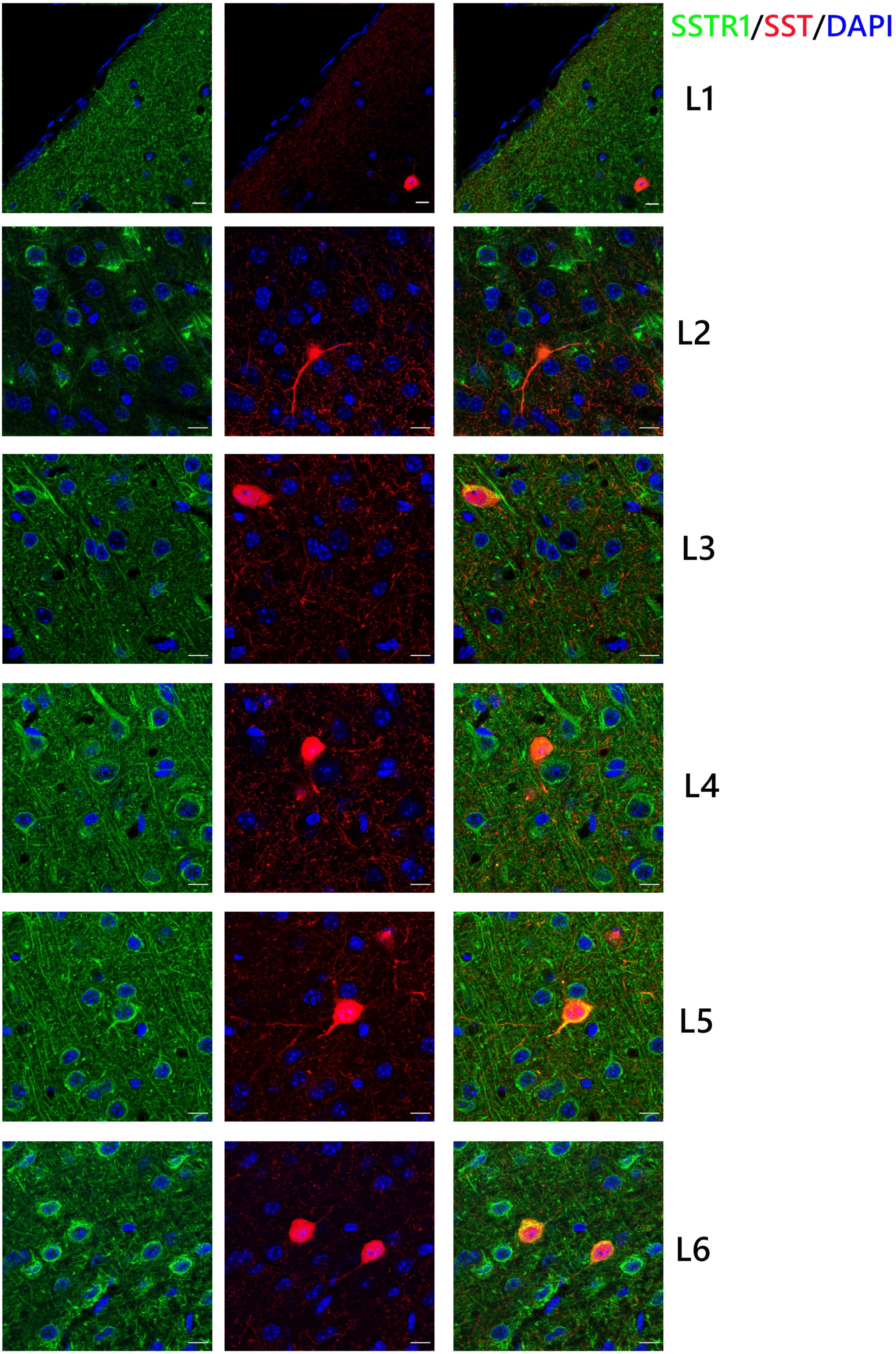

SSTR1/SST/DAPI
L1
L2
L3
L4
L5
L6

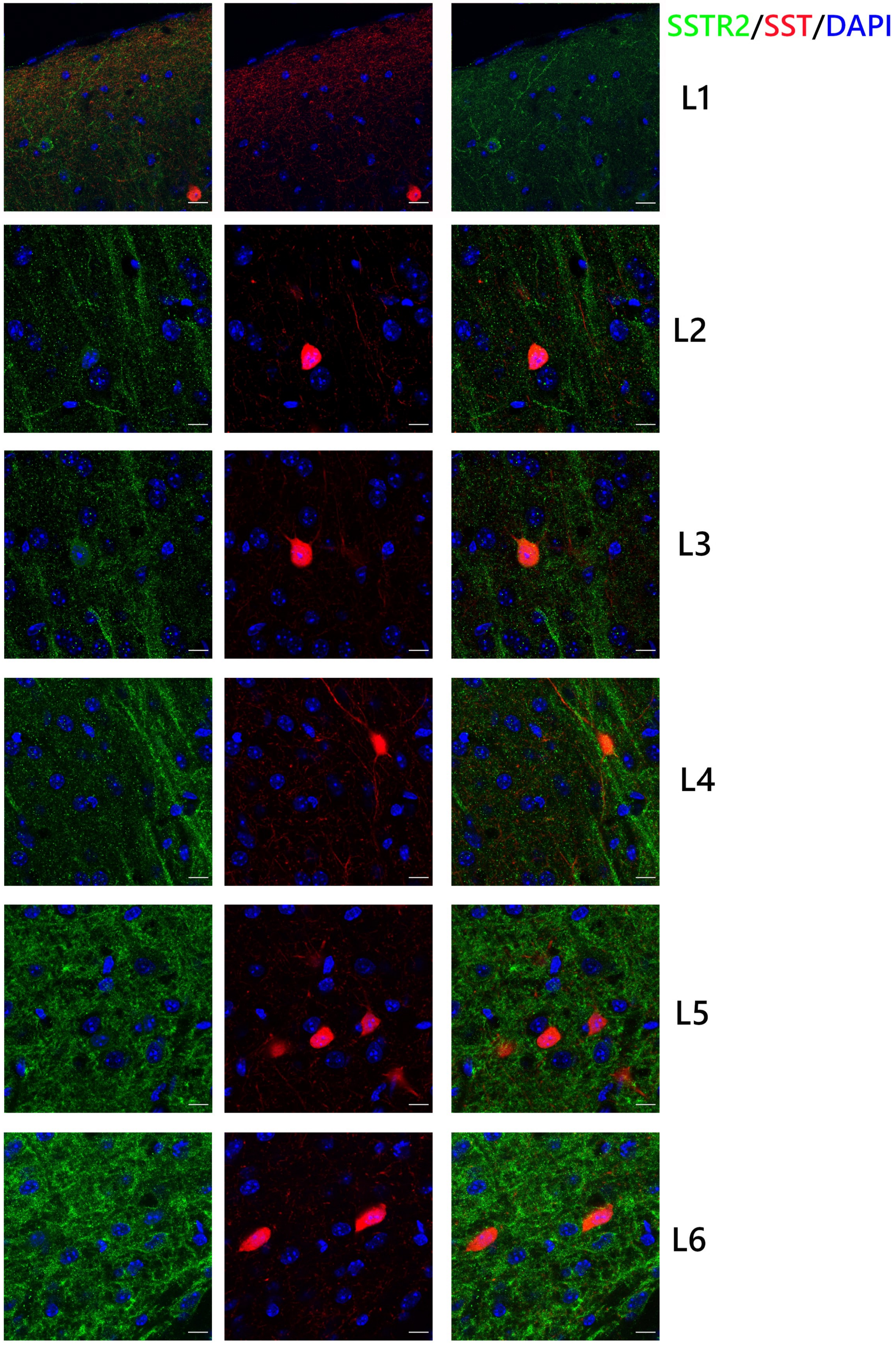
SSTR2/SST/DAPI
L1
L2
L3
L4
L5
L6

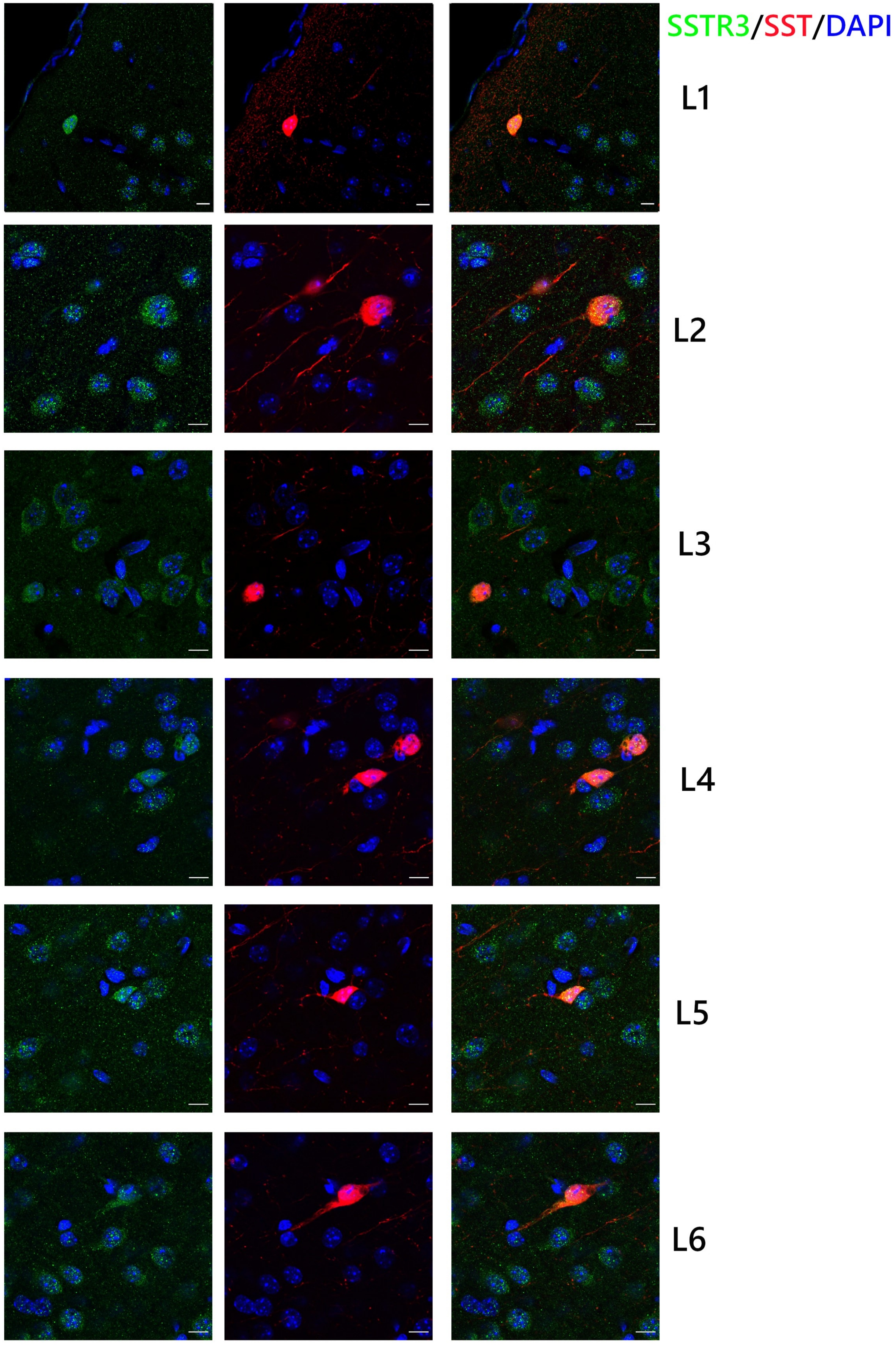
SSTR3/SST/DAPI
L1
L2
L3
L4
L5
L6

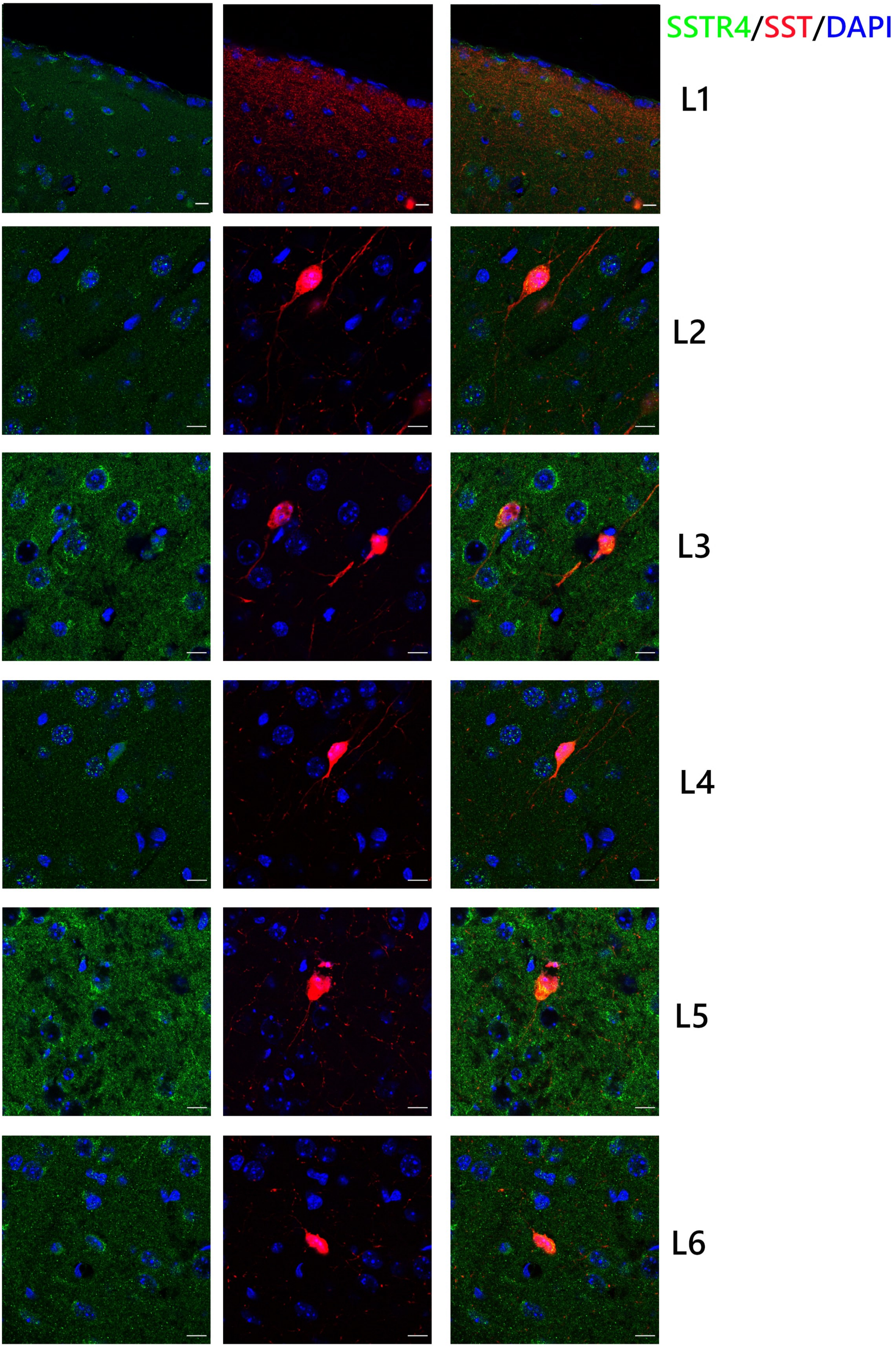
SSTR4/SST/DAPI
L1
L2
L3
L4
L5
L6

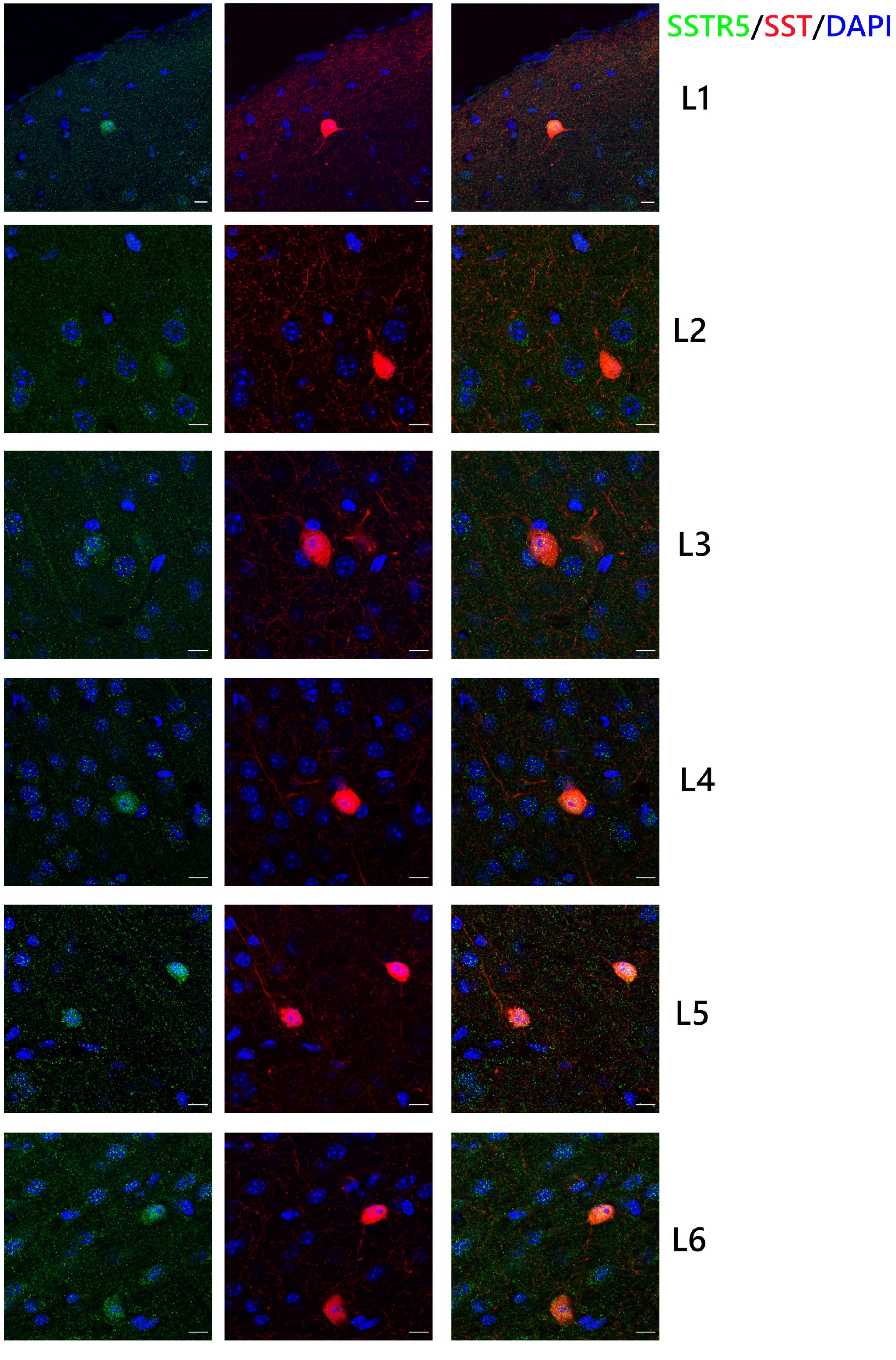
SSTR5/SST/DAPI
L1
L2
L3
L4
L5
L6

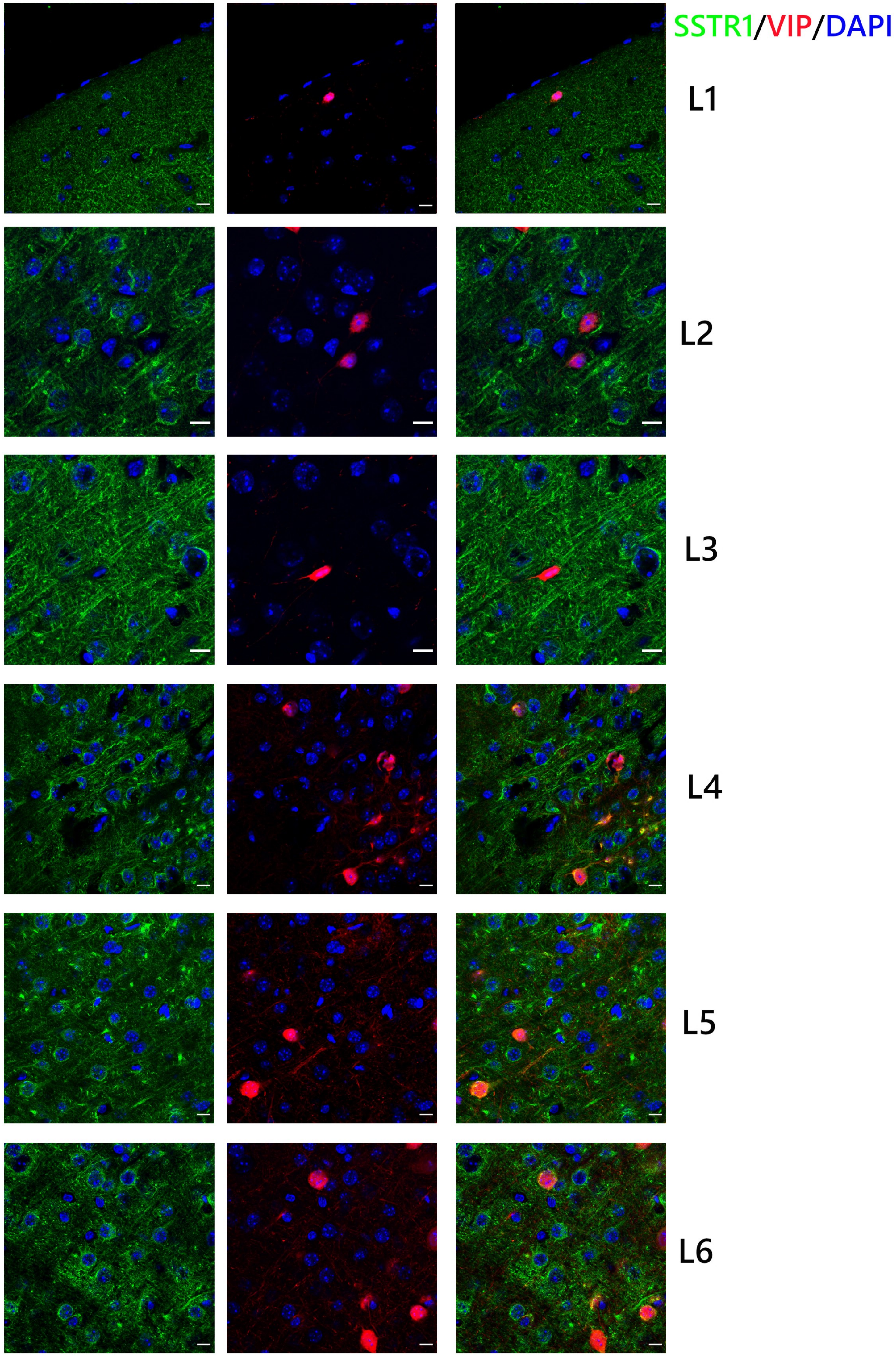
SSTR1/VIP/DAPI
L1
L2
L3
L4
L5
L6

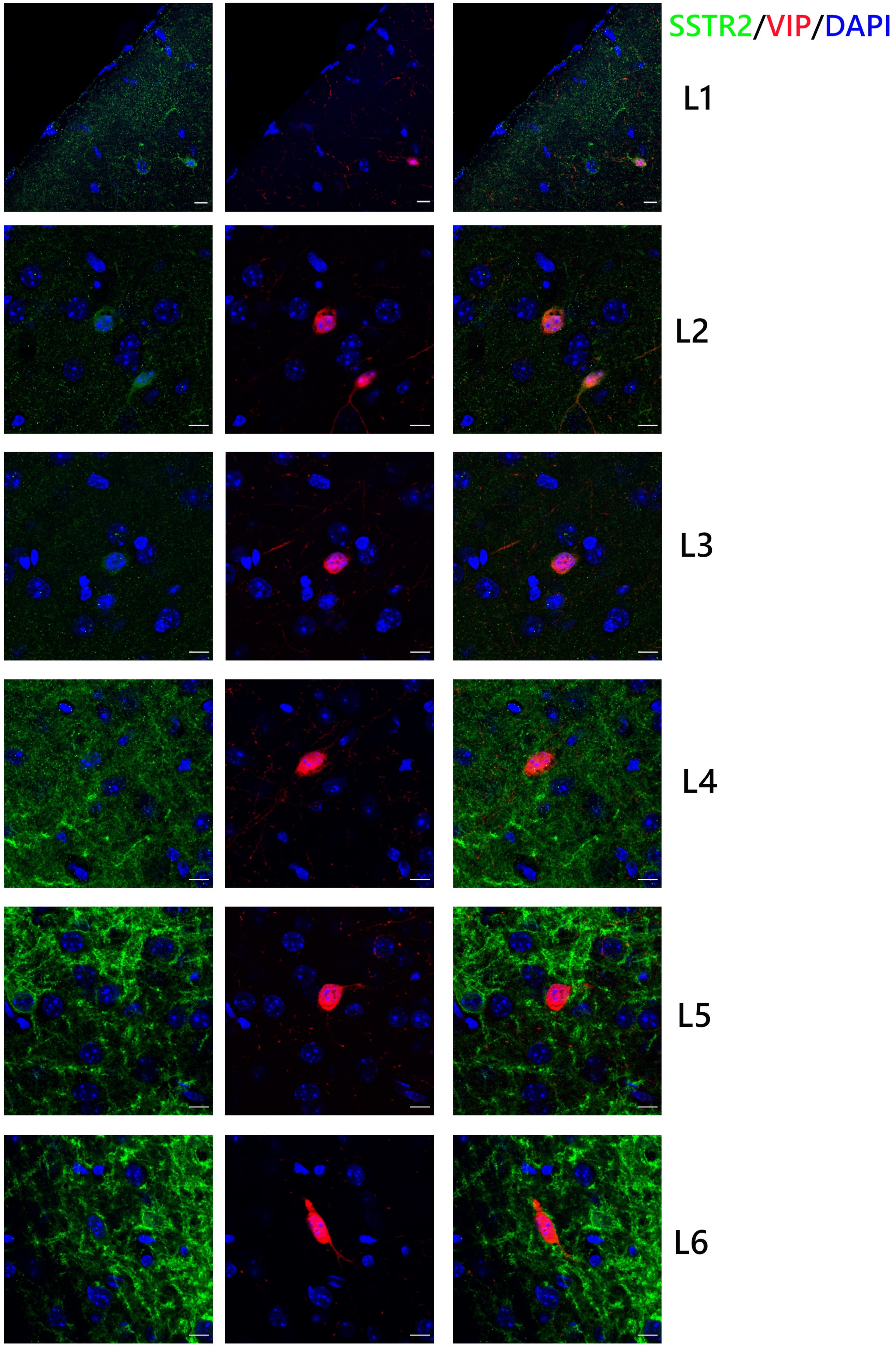

SSTR2/VIP/DAPI
L1
L2
L3
L4
L5
L6

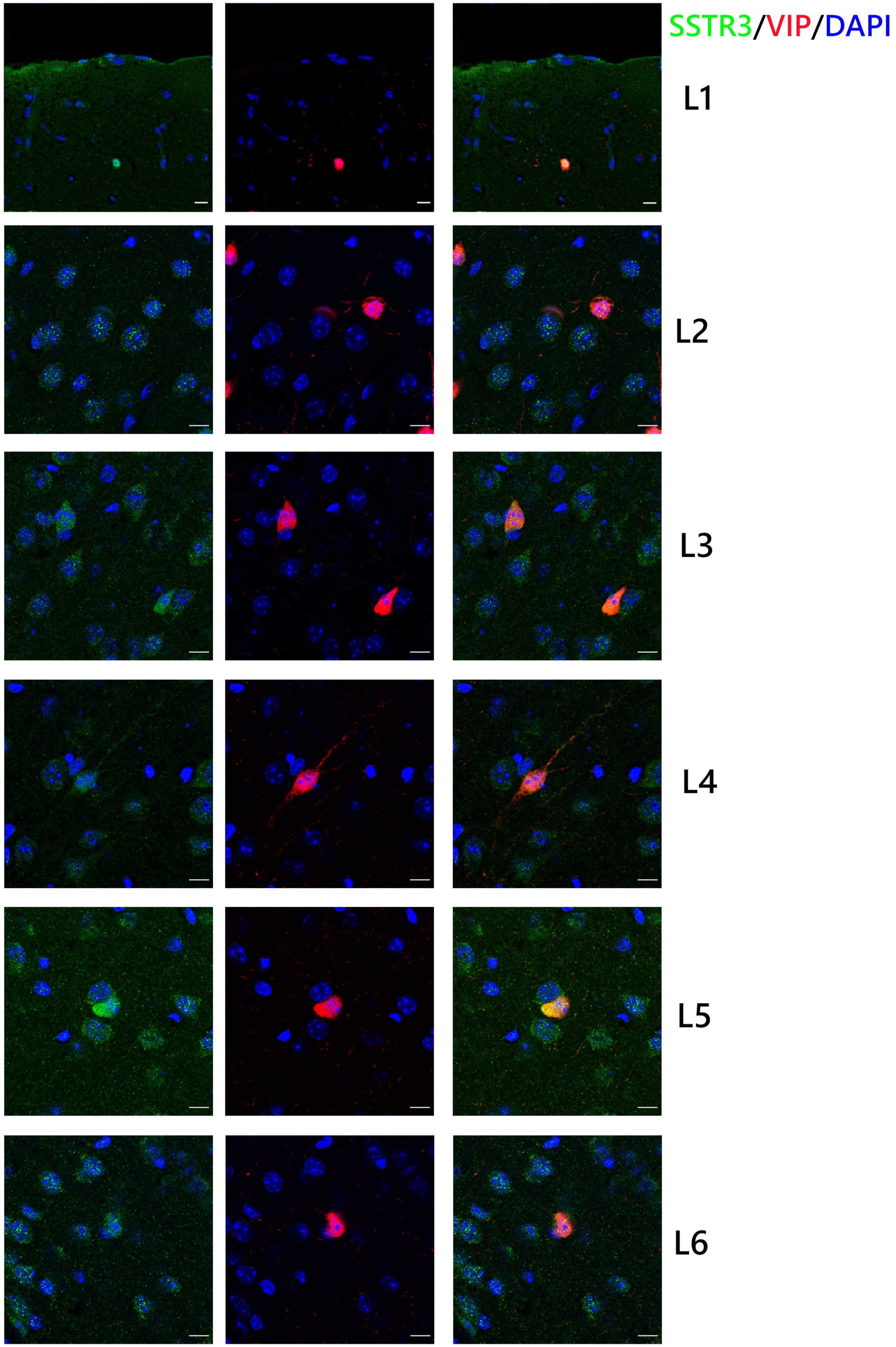

SSTR3/VIP/DAPI
L1
L2
L3
L4
L5
L6

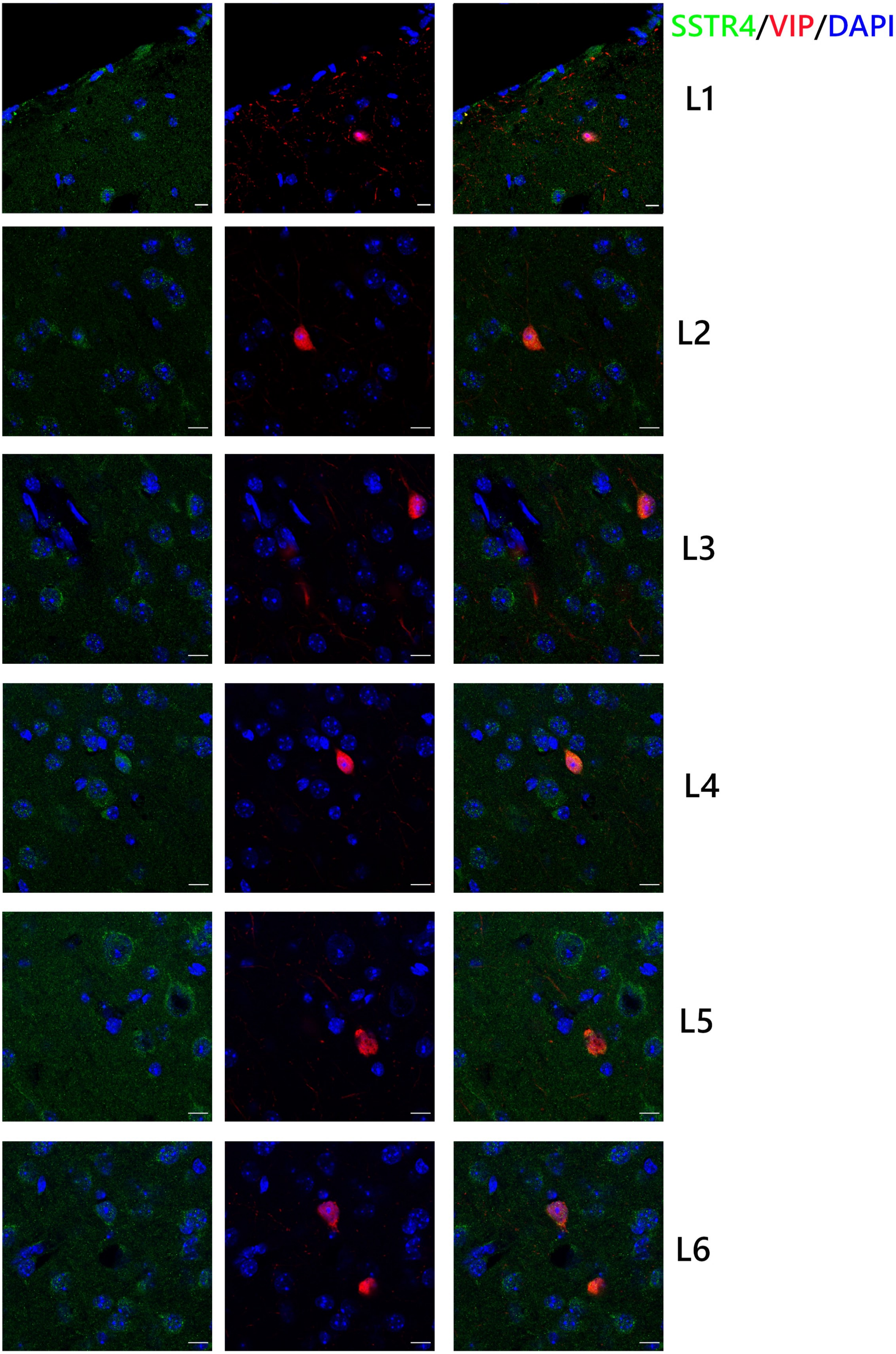
SSTR4/VIP/DAPI
L1
L2
L3
L4
L5
L6

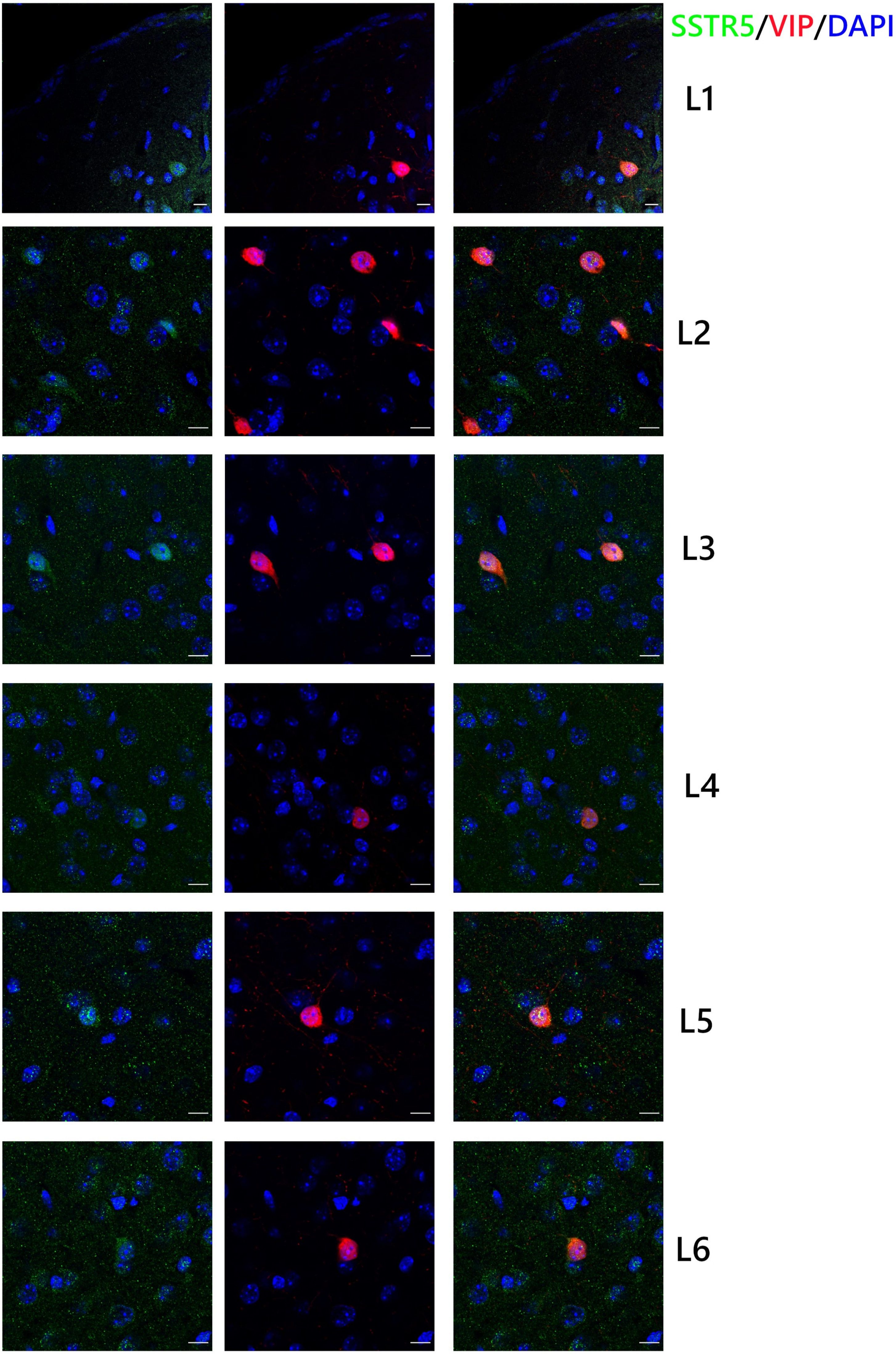
SSTR5/VIP/DAPI
L1
L2
L3
L4
L5
L6

Table S1. Table presents results of quantitative analysis of the density of particular INs and percentage of SSTR immunopositive INs in different cortical layers of mouse somatosensory cortex. Mean ± SD.

	PV INs/mm^2	SSTR positive PV INs/mm^2	Percentage of SSTR positive PV INs	SST INs/mm^2	SSTR positive SST INs/mm^2	Percentage of SSTR positive SST INs	VIP INs/mm^2	SSTR positive VIP INs/mm^2	Percentage of SSTR positive VIP INs
**L2/3**									
**SSTR1**	316.9 ± 24.4	243.9 ± 13.8	77.4 ± 8.7	80.9 ± 9.2	52.4 ± 6.2	65.9 ± 13.5	98.9 ± 31.8	28.4 ± 12.5	28.6 ± 7.3
**SSTR2**	-	-	-	76.0 ± 10.5	28.1 ± 10.1	36.4 ± 8.8	106.1 ± 31.3	62.9 ± 29.0	57.0 ± 10.3
**SSTR3**	209.1 ± 60.0	158.7 ± 63.2	74.3 ± 10.3	103.1 ± 21.6	85.0 ± 24.6	81.4 ± 7.0	125.0 ± 26.2	108.7 ± 23.5	87.0 ± 3.9
**SSTR4**	151.1 ± 55.7	64.3 ± 37.6	40.2 ± 11.8	85.3 ± 15.4	57.2 ± 16.8	66.1 ± 10.1	103.7 ± 27.9	57.9 ± 18.0	55.6 ± 5.1
**SSTR5**	146.6 ± 20.8	109.0 ± 19.8	74.1 ± 5.2	87.8 ± 20.6	78.8 ± 19.8	89.9 ± 7.8	113.2 ± 16.9	91.9 ± 21.2	80.5 ± 8.6
**L4**									
**SSTR1**	215.0 ± 31.0	165.6 ± 31.5	76.9 ± 7.2	124.4 ± 27.3	91.8 ± 19.8	74.3 ± 10.8	93.6 ± 47.5	44.7 ± 21.0	48.9 ± 6.8
**SSTR2**	-	-	-	156.4 ± 59.0	54.4 ± 22.9	35.2 ± 5.7	88.0 ± 14.9	37.9 ± 15.7	42.8 ± 15.0
**SSTR3**	290.6 ± 50.7	260.9 ± 45.1	89.9 ± 5.7	234.0 ± 29.3	212.5 ± 24.3	91.0 ± 5.2	77.3 ± 10.2	66.9 ± 14.3	85.8 ± 9.9
**SSTR4**	335.1 ± 23.9	205.8 ± 32.0	62.1 ± 13.7	181.4 ± 28.1	110.5 ± 22.4	60.9 ± 7.6	69.9 ± 9.9	40.8 ± 6.9	59.0 ± 11.2
**SSTR5**	312.8 ± 36.4	235.5 ± 31.5	75.3 ± 7.0	205.3 ± 71.3	168.0 ± 53.8	83.0 ± 5.1	63.1 ± 11.4	56.7 ± 9.4	90.5 ± 9.2
**L5/6**									
**SSTR1**	220.5 ± 40.5	189.8 ± 48.9	77.4 ± 8.7	250.1 ± 42.4	234.0 ± 42.8	93.4 ± 3.5	25.6 ± 10.4	13.8 ± 5.5	54.1 ± 3.8
**SSTR2**	-	-	-	215.3 ± 46.5	36.7 ± 19.1	16.8 ± 6.7	38.2 ± 9.9	18.4 ± 6.4	47.1 ± 6.1
**SSTR3**	214.2 ± 63.0	206.0 ± 61.4	96.2 ± 2.4	231.0 ± 14.0	224.6 ± 12.7	97.3 ± 1.9	34.3 ± 5.5	29.6 ± 5.8	85.9 ± 5.1
**SSTR4**	132.6 ± 30.2	48.8 ± 17.6	35.8 ± 6.6	171.9 ± 28.1	83.1 ± 22.5	48.9 ± 11.9	28.9 ± 11.0	9.0 ± 4.9	30.1 ± 7.8
**SSTR5**	175.5 ± 74.4	162.8 ± 65.0	93.0 ± 7.0	221.5 ± 32.9	201.7 ± 43.6	90.4 ± 7.6	28.1 ± 5.6	24.1 ± 6.3	85.4 ± 13.4
